# Compound Annotation by UHPLC-MS/MS, Quantification of Phenolic Compounds and Antimicrobial Activity of Monofloral Avocado Honey

**DOI:** 10.3390/plants14213340

**Published:** 2025-10-31

**Authors:** Tom E. C. Sarmento, Veronica de M. Sacramento, Murilo M. Brandão, Afrânio F. de Melo Júnior, Elytania V. Menezes, Pedro H. F. Veloso, Nathália da C. Pires, Carlos H. G. Martins, Gabriel G. Caléfi, Tânia M. A. Alves, Alisson S. P. Caldeira, Dario A. de Oliveira, Vanessa de A. Royo

**Affiliations:** 1 Laboratory of Natural Products, Department of General Biology, State University of Montes Claros, Montes Claros 39401-089, MG, Brazil; eumesmoquem35@gmail.com (T.E.C.S.); veronica.sacramento.2014@gmail.com (V.d.M.S.); pedrofonsecambc@gmail.com (P.H.F.V.); 2 Laboratory of Bioprospecting and Genetic Resources, Department of General Biology, State University of Montes Claros, Montes Claros 39401-089, MG, Brazil; murilomalveira@yahoo.com.br (M.M.B.); afraniofariasdemelo@gmail.com (A.F.d.M.J.); menezes.elytania@gmail.com (E.V.M.); 3 Bioprocess Development Laboratory (Multiuser), State University of Montes Claros, Montes Claros 39401-089, MG, Brazil; dario.aol@gmail.com; 4 Cooperative of Beekeepers and Family Farmers of Northern Minas, Fazenda Bahia s/n, Bocaiuva 39390-000, MG, Brazil; nathaliaengali@gmail.com; 5 Laboratory of Microbiology, Institute of Biomedical Sciences, Department of Microbiology, Federal University of Uberlândia, Uberlândia 38408-100, MG, Brazil; carlos.martins2@ufu.br (C.H.G.M.); ggcalefi@ufu.br (G.G.C.); 6 Laboratory of Chemistry of Natural Bioactive Products, René Rachou Institute-Fiocruz Minas, Belo Horizonte 30190-002, MG, Brazil; tania.alves@fiocruz.br (T.M.A.A.); alisson.caldeira@fiocruz.br (A.S.P.C.); 7 Center for Technology and Innovation in Bioeconomy, Department of General Biology, State University of Montes Claros, Alterosa District, Bocaiuva 39390-000, MG, Brazil

**Keywords:** avocado honey, monofloral honey, antioxidant activity, phenolic compounds, LC-MS/MS, bioactive metabolites, nutritional value, therapeutic potential

## Abstract

Honey is a natural product of high nutritional and therapeutic value, whose biological properties are closely linked to its botanical origin and chemical composition. This study aimed to characterize avocado honey in terms of botanical origin, physicochemical parameters, phenolic content, antioxidant activity, chemical profile by LC-MS/MS, and antibacterial potential. Melissopalynological analysis revealed 86.21% avocado pollen, allowing classification as monofloral honey. The sample presented amber color and a high total phenolic content (269.79 ± 1.10 mg GAE 100 g^−1^), values higher than those commonly reported for Brazilian and international honeys. Antioxidant activity, assessed by the DPPH method, confirmed the strong radical-scavenging capacity, consistent with the phenolic profile identified (EC_50_ 10.250 ± 0.003 mg mL^−1^). LC-MS/MS analysis allowed the annotation of nine compounds, including caffeine, scopoletin, abscisic acid, and vomifoliol, compounds associated with antioxidant, anti-inflammatory, and metabolic regulatory activities. Although no antibacterial effect was detected against the tested oral bacterial strains, the results highlight the chemical diversity and functional potential of avocado honey. Overall, the findings reinforce the bioactive potential of avocado honey, particularly due to its strong antioxidant capacity and diversity of metabolites, supporting its value as a natural resource of nutritional and therapeutic interest.

## 1. Introduction

Beekeeping has great potential in Brazil, as the country’s territory harbors a high diversity of flora and provides favorable climatic conditions for several honey-producing bee species. It is an agricultural activity that meets all three requirements of sustainability—economic, social, and ecological—since it provides income for beekeepers, generates employment opportunities, and contributes to the preservation of native flora. Moreover, it yields honey, pollen, propolis, and wax [1]. Analyses of different types of honey are also of great importance in the global market, as the trade of honey-derived products has grown considerably in recent years. The main reason for honey use in foods is its rich nutritional value and additional non-nutritional functionalities [2].

Honey is a natural, sweet substance produced by bees from the nectar of flowers or from secretions of living parts of plants or excretions of plant-sucking insects. After processing by specific enzymes, it is stored in honeycombs for future bee nutrition [3,4]. It is characterized as a natural product with a complex composition, being predominantly made up of sugars, especially glucose and fructose (60–85%). In smaller proportions, it contains proteins (0.2–1.6%) and various amino acids, such as alanine, phenylalanine, glutamic acid, leucine, tyrosine, and proline. Its matrix also includes important enzymes, such as invertase, catalase, acid phosphatase, diastase, and glucose oxidase. In addition to these components, honey contains phenolic compounds, including phenolic acids and flavonoids, essential minerals (sodium, magnesium, calcium, and iron), and vitamins, particularly B-complex vitamins and vitamin C [5,6,7,8]. Several environmental factors, such as floral origin, soil, climate, and geographical location, also directly influence its composition and quality. Among these, secondary metabolites such as phenolic compounds are especially important, as they are transferred from plants to honey by bee activity [9,10,11]. The identification of phenolic acids and flavonoids associated with floral sources can add commercial value by certifying botanical origin and linking it to specific chemical and biological properties.

Although mainly consumed as food due to its high sugar content and nutritional value, honey has also been widely used in traditional medicine, both orally and topically, against various diseases. Its therapeutic properties include antimicrobial, antioxidant, gastroprotective, anticancer, anti-inflammatory, and immunomodulatory activities [12,13,14,15,16].

Honey can also be classified as monofloral or polyfloral, depending on the amount of nectar from each plant used for its production. Another classification is based on the raw material: it is considered honeydew honey when the material comes from secretions of plant-sucking insects or from living parts of plants, and floral honey when nectar is the main source [3]. It is now well established that the botanical origin of honey strongly influences its phenolic content and, therefore, its antioxidant activity [17]. This explains why certain monofloral honeys with high antioxidant activity, such as aroeira honey, may reach higher market value. Other factors, such as raw material sources or seasonality, also alter honey’s complex composition, providing greater diversity of physicochemical properties and health benefits [18].

The health benefits of honey are attributed to a wide variety of bioactive compounds, with more than 200 substances identified across different classes of secondary metabolites, such as phenolic compounds, organic acids, vitamins, and proteins [19,20]. In addition to sugars that provide its sweet taste, the high osmolarity of honey contributes to bacterial growth inhibition by withdrawing water essential for bacterial development, combined with its naturally low water content (15–21%). Another factor in pathogen inhibition is pH, since the undissociated form of weak acids can freely cross bacterial cell walls, acidify the cytoplasm, and denature proteins [21]. Some honeys present pH values as low as 4.0. These antibacterial properties allow honey to be classified as an antimicrobial agent [12].

Dental caries is one of the most common chronic diseases worldwide, affecting around 35% of the global population [22]. The main strategy to prevent caries is to reduce or eliminate cariogenic bacteria such as *Streptococcus mutans*, often controlled with detergents such as sodium lauryl sulfate, sodium lauroyl sarcosinate, and cocamidopropyl betaine in toothpastes [23]. However, detergents may cause cytotoxic or allergic effects in some individuals, prompting interest in organic and natural alternatives [24].

Thus, honey emerges as a promising tool for controlling cariogenic bacteria. In addition to the difficulty bacteria face in developing resistance against honey’s antimicrobial characteristics, it is also effective against antibiotic-resistant strains [25].

Several chromatographic methods can be used to analyze honey composition, the most common being liquid chromatography (HPLC), which can be coupled with mass spectrometry for more accurate analysis [26]. This analytical process is highly important as it allows the detection of substances at extremely low concentrations, enabling the identification of compounds present in honey, the discovery of novel bioactive substances, and the elucidation of the origins of various physicochemical characteristics. It also helps ensure purity and quality, since contaminants may compromise honey’s beneficial effects [27]. Due to these possibilities, there has been growing interest in the development of natural-source products, as they offer lower costs and fewer side effects [28]. Consequently, the number of studies on the composition and properties of different types of honey has increased significantly, adding commercial value to honeys previously overlooked in the market [29].

The present study explored melissopalynological analysis, phenolic and total flavonoid content, antioxidant capacity, and chromatographic profile of avocado (*Persea americana* Mill.) honey, evaluated its antimicrobial activity against cariogenic bacteria, and identified substances with potential bioactive effects.

## 2. Results

### 2.1. Botanical Identification

Pollen analysis of honey, whether qualitative or quantitative, allows the observation of pollen grains and the pollen spectrum, indicating the plants visited by bees and enabling the characterization of honey based on its botanical origin [30,31]. Honeys can be classified as heterofloral or wild, originating from the nectar of several plant species, and as monofloral or unifloral, which are the most commercially valued. The floral origin of honey can be determined by recognizing the dominant pollen grains [32]. Pollen grains from different species are grouped according to relative frequencies, being considered dominant when they exceed 45% of the count, accessory when ranging from 15% to 45%, and isolated when occurring at lower frequencies [33].

In the analyzed sample, 86% of avocado pollen was observed, classifying the honey as predominantly floral. In addition, pollens from other plant species such as *Cissus verticillata* (cipó-uva), *Eucalyptus* sp. and *Myracrodruon urundeuva* (aroeira) were identified in much lower concentrations (2.15–6.56%) (Table 1). Therefore, the identification of pollen morphology assists not only in determining the botanical species visited by bees, but also in assessing the intensity of visits, the geographical origin of the honey, and its classification as monofloral, predominant, or polyfloral. In the analyzed sample, 86% of avocado pollen was observed, classifying the honey as predominantly floral. In addition, pollens from other plant species such as *Cissus verticillata* (cipó-uva), *Eucalyptus* sp. and *Myracrodruon urundeuva* (aroeira) were identified in much lower concentrations (2.15–6.56%) (Table 1). Therefore, the identification of pollen morphology assists not only in determining the botanical species visited by bees, but also in assessing the intensity of visits, the geographical origin of the honey, and its classification as monofloral, predominant, or polyfloral.

### 2.2. Determination of the Honey Color

The color of the honey was determined following the Pfund scale [34]. The spectrophotometric reading for the avocado honey sample was 0.321 absorbance, which corresponds approximately to 41 mm on the Pfund scale, classifying the sample as amber.

### 2.3. Total Polyphenols

The total polyphenol levels were determined by the Folin–Ciocalteu method, read at 765 nm, with R^2^ = 0.994 determined on the gallic acid standard curve. It can be observed that the value found for the analyzed honey was 269.79 ± 1.10 mgGAE 100 g^−1^.

### 2.4. Antiradical Capacity

In determining the antioxidant capacity, the test with 2,2-diphenyl-1-picrylhydrazyl (DPPH) was used. To determine EC_50_ in mg mL^−1^, the equations of the standard lines for gallic acid and for the honey were used. The R^2^ of the equations were between 0.983 and 0.992. The result observed for avocado honey was EC_50_ 10.250 ± 0.003 mg mL^−1^ and EC_50_ 3.01 ± 0.01 µg mL^−1^ for gallic acid.

### 2.5. LC/MS/MS Analysis

Following elution of the avocado honey sample, various compounds were detected between 6.2 and 22.9 min, based on exact mass and characteristic fragmentation patterns using UHPLC-MS/MS in positive ionization mode (Figure S1). Using this method, ten substances with distinct masses and molecular formulas were identified and are summarized in Table 2.

### 2.6. Antibacterial Activity Assay

The assays for MIC determination showed no change in bacterial growth of the strains *S. faecalis*, *S. mitis*, *L. paracasei*, *S. mutans*, *S. sobrinus*, *S. sanguinis* and *S. salivarius*, even at the highest concentration of the honey extract (20%). The same was observed in the MBC assays (Table 3).

## 3. Discussion

### 3.1. Botanical Identification

The honey was subjected to qualitative and quantitative microscopic analysis, based on the identification of the species of each pollen grain present and their respective quantities in the sample. This approach also allows the assessment of the floral diversity of the region and the inference of local beekeeping practices [46] enabling the determination of which plants were visited by bees, which plants were most frequently visited during honey production, and allowing the classification of botanical origin [31].

Honeys can be classified as polyfloral or wild when they are produced from the nectar of multiple plant species. When there is a higher concentration of pollen from a single species, they can be classified as predominant or monofloral, with pollen concentrations exceeding 90%, or, according to recent criteria, above 45% for botanical authentication purposes [47]. Higher pollen concentration from a single species is more appealing to consumers and adds commercial value [48].

The analyzed honey presented 86.21% avocado pollen (Table 1), which approaches the classification of monofloral honey and allows inferring that most of its physicochemical and bioactive characteristics, such as antioxidants and specific sugars, predominantly derive from avocado nectar [47]. This concentration is highly relevant, as it reflects dominant flowering and facilitates honey standardization and quality control, also enabling the use of specific chemical markers, such as perseitol, for the authentication of monofloral avocado honey [47].

Therefore, the combination of pollen and chemical analyses provides a robust approach for the characterization and authentication of avocado honey, ensuring its quality and origin for consumers.

### 3.2. Determination of the Honey Color

The avocado honey was classified as amber, according to the Pfund scale. Honey color is closely related to its antioxidant activity, which tends to increase proportionally with color intensity, possibly due to the presence of compounds such as anthocyanins and flavones [49]. In addition to chemical composition, factors such as botanical origin, climatic conditions during nectar secretion, and maturation temperature within the hive also influence honey color [50]. In general, darker honeys are characterized by higher mineral content and elevated levels of calcium, iron, B-complex vitamins, and vitamin C, as well as a more pronounced aroma, whereas lighter honeys tend to have higher sodium levels [51,52].

Studies conducted with samples from northern Minas Gerais showed that aroeira honeys were predominantly classified as dark amber, while pequi and betônica honeys corresponded to amber, velame honeys to light amber, and cipó-uva honeys ranged from extra-light amber [12] to dark amber [17]. This variability within the same species may be associated with multiple factors, including pollen composition, which plays an important role in determining honey color [17].

### 3.3. Total Polyphenols

The phenolic content found in the avocado honey sample studied was 269.79 ± 1.10 mg GAE 100 g^−1^. This result is higher than that of Brazilian monofloral and multifloral honeys investigated in Northern Minas Gerais, in which the quantified samples showed phenolic compounds ranging from 42.52 ± 3.29 mg GAE 100 g^−1^ to 107.93 ± 1.12 mg GAE 100 g^−1^ [17] and from 40.70 ± 0.03 mg GAE 100 g^−1^ to 84.77 ± 0.05 mg GAE 100 g^−1^ [12]. In a more recent quantification, the monofloral avocado honey sample corresponded to 124.07 mg GAE 100 g^−1^ [53], still lower than the present value. Other studies on the phenolic quantification of honeys, including avocado samples, were carried out in Ecuador and Spain, with the respective values being 68.23 ± 5.79 mg GAE 100 g^−1^ and 117 ± 2.74 mg GAE 100 g^−1^ [54,55].

The therapeutic potential of honey, especially related to antimicrobial, anti-inflammatory, anticarcinogenic, and antioxidant effects, has been highlighted in scientific investigations over the last decades. The different classes of substances present in honey that exhibit antioxidant activity are generally phenolic compounds, which may act synergistically [56,57].

Antioxidant molecules can interact and inhibit the initiation or propagation of oxidation chain reactions generated by reactive free radicals before vital molecules are damaged. The neutralization of free radicals can provide organic systems with an overall reduction in diseases associated with oxidative stress, such as neurodegenerative processes, cardiovascular diseases, liver disorders, and other pathologies [4,57].

The properties attributed to the honey constituents, particularly phenolic compounds, depend on the plant species; the geographic and botanical origin of the honey sample and the climatic conditions are important factors, especially when considering the quality of secondary metabolites that honey inherits from its plant sources [3,58,59]. This distinct chemical composition and properties likely exert unique biological effects and justify the different health outcomes observed, thus contributing to the mixed nature of the results [59].

### 3.4. Antiradical Capacity

The DPPH method was used to determine the antioxidant behavior of the avocado honey sample. In this assay, the result was expressed as EC_50_, which corresponds to the concentration of the sample able to inhibit 50% of a free radical. The result was compared with a gallic acid solution. For avocado honey EC_50_ 10.250 ± 0.003 mg mL^−1^. Among the monofloral honey samples investigated in Northern Minas Gerais, EC_50_ values ranged from 51.48 ± 1.48 mg mL^−1^ to 150.71 ± 2.56 mg mL^−1^ [12] and from 11.30 ± 0.05 mg mL^−1^ to 62.12 ± 0.13 mg mL^−1^ [17]. In a study in which 15 honeys from Spain were investigated, including both polyfloral and monofloral types, the antioxidant capacity of the honey samples varied with EC_50_ values ranging from 5.46 ± 0.05 to 202 ± 5.53 mg mL^−1^, and the avocado honey sample corresponded to 13.8 ± 0.07 mg mL^−1^, which corroborates the value obtained in this study and is consistent with the chromatographic profile, in which no remarkable differences were observed in the composition of this honey regarding phenolic acids and flavonoids [55].

It is noteworthy, as already observed in another study, that radical scavenging activity was different not only among different honey samples within the same region but also in relation to samples from other countries [60].

In honey, chemical complexity results from the presence of different classes of compounds, including alcohols, aldehydes, acids, ketones, terpenes, norisoprenoids, benzene-derived aromatic compounds, hydrocarbons, and others [47,55], as also noted in this investigation. Such constituents contribute to the characteristic aromatic profile of honey and can be used as chemical markers for the determination of botanical and geographical origin [60]. Although present only in low concentrations, these different classes of compounds may contribute to the therapeutic activities of honey, particularly to the antioxidant effect, which is associated with the natural potential of honey to scavenge free radicals [61].

Antioxidant activity, at least in part, may contribute to the anti-inflammatory action, since reactive oxygen species are involved in different processes associated with inflammation [60].

### 3.5. LC/MS/MS Analysis

The annotation of nine compounds was carried out based on the accurate mass and characteristic fragmentation patterns detected in honey by liquid chromatography coupled with mass spectrometry (LC-MS), allowing the putative assignment of metabolites present in the matrix. These findings provided the basis for the comparative analysis and discussion regarding the chemical composition and the biological potential of the sample.

2-Hydroxyquinoline, molecular formula C_9_H_7_NO, was annotated from the major protonated ion [M+H]^+^ at *m*/*z* 146.0589, with characteristic fragments at *m*/*z* 118.0620 and *m*/*z* 117.0552 [35]. This compound was reported in aroeira honey from Northern Minas Gerais, Brazil [35]. 2-Hydroxyquinoline and its analogues have been associated with the inhibition of α-amylase and α-glucosidase enzymes, contributing to the maintenance of stable glycemia and potentially serving as an adjuvant in diabetes management [62]. Although 2-hydroxyquinoline has not been extensively studied, it is hypothesized to exhibit chelating potential similar to its isomer 8-hydroxyquinoline, which has been investigated for neuroprotective, anti-inflammatory, anticancer, and antimicrobial properties, suggesting additional biological activities [63].

Caffeine, molecular formula C_10_H_8_O_4_, was annotated from the major protonated ion [M+H]^+^ at *m*/*z* 195.0879, with characteristic fragments at *m*/*z* 138.0648, corresponding to the loss of methyl isocyanate [M+H–O=C=NCH_3_] through a retro-Diels–Alder rearrangement, and at *m*/*z* 110.0720, derived from an additional CO loss [36]. Caffeine has been identified in monofloral coffee honeys from Vietnam [64], Espírito Santo, Brazil [65], and in betônica, coffee, cipó-uva, and pequi honeys from Northern Minas Gerais [35]. Caffeine is a well-known alkaloid with stimulant activity, neuroprotective properties [66], cognitive-enhancing effects [67], and recognized as an ergogenic aid improving physical performance across various sports [68].

Scopoletin, molecular formula C_10_H_8_O_4_, was annotated from the main protonated ion [M+H]^+^ at *m*/*z* 193.0497, with characteristic fragments at *m*/*z* 177.0549 ([M+H–CH_3_]^+^, demethylation), *m*/*z* 165.0545 ([M+H–CO]^+^, loss of CO), and *m*/*z* 137.0581 ([M+H–2CO]^+^) [37,38]. Scopoletin has been detected in cotton honey [37], oak honey from Greece [69] and in betônica, coffee, and velame honeys from Northern Minas Gerais [35]. As a coumarin, scopoletin is documented for its antifungal, antibacterial, antioxidant, antihypertensive, antidiabetic, hepatoprotective, and neuroprotective activities [70,71]. Its anti-inflammatory effect has been confirmed both in vitro and in vivo, showing inhibition of pro-inflammatory cytokines such as TNF-α, IL-1β, IL-12, IL-6, PGE2, COX-2, and iNOS in various cell and animal models [72,73].

Abscisic acid (ABA) and/or its isomers, molecular formula C_15_H_20_O_4_, were annotated from the major protonated ion [M+H]^+^ at *m*/*z* 265.1441, with characteristic fragments at *m*/*z* 247.1334 ([M+H–H_2_O]^+^), *m*/*z* 229.1222 ([M+H–2H_2_O]^+^), *m*/*z* 201.1284 ([M+H–2H_2_O–CO]^+^), and *m*/*z* 153.0906 [39,42]. ABA has been consistently reported in honeys of different floral origins: acacia, linden, chestnut, fir, and multifloral Slovenian honeys [74]; citrus, clover, alfalfa, buckwheat, and multifloral American honeys [42]; dill honey [41]; and manuka honey [45]. It has also been detected in Greek monofloral honeys, particularly oak honey [69] and in aroeira, coffee, cipó-uva, pequi, betônica, and velame honeys from Northern Minas Gerais [35]. ABA is a protective phytohormone involved in fruit ripening, leaf senescence, seed dormancy, and plant responses to abiotic stress such as extreme temperatures, heavy metal toxicity, and parasitic interactions [75,76,77]. It has also been linked to hypoglycemic effects, improving glucose tolerance in diabetic patients [78], protective and therapeutic effects against inflammatory bowel disease [79], lung inflammation and oxidative stress [40], and influenza infection [80]. Additionally, ABA has been associated with malaria modulation in both mammalian hosts and insect vectors, reducing parasitemia and transmission [81].

Tuberonic acid (TA), molecular formula C_12_H_18_O_4_, was annotated from the main protonated ion [M+H]^+^ at *m*/*z* 227.1286, with characteristic fragments at *m*/*z* 209.1179 ([M+H–H_2_O]^+^), *m*/*z* 191.1069 ([M+H–2H_2_O]^+^), *m*/*z* 163.1110 ([M+H–2H_2_O–CO]^+^) [43,44], as well as *m*/*z* 149.0950 ([M+H–2H_2_O–C_2_H_2_O]^+^) and *m*/*z* 131.0839 ([M+H–3H_2_O–C_2_H_2_O]^+^) [35]. TA has been reported in stingless bee emerald honey from southern Bangkok [82] and in coffee, velame, and multifloral honeys from Northern Minas Gerais [35]. TA belongs to the jasmonate class, derivatives of jasmonic acid that play crucial roles in regulating physiological processes and mediating developmental and adaptive responses in plants [83]. It influences the activity of jasmonoyl-isoleucine, the active jasmonate hormone in stress signaling, modulating plant defenses [84]. In mice fecal metabolite studies, TA was linked to anti-inflammatory activity through inhibition of pro-inflammatory cytokines, promoting intestinal healing and restoration of the mucosal barrier in colitis models. It has also shown antioxidant activity, contributing to the reduction of oxidative stress in inflamed tissues, positioning TA as a promising therapeutic candidate for inflammatory bowel diseases [43].

Dihydroconiferin, molecular formula C_16_H_24_O_8_, was annotated from the main protonated ion [M+H]^+^ at *m*/*z* 345.1551, with characteristic fragments at *m*/*z* 183.1019 ([M+H–C_6_H_11_O_5_]^+^), *m*/*z* 165.0913 ([M+H–C_9_H_11_O_6_]^+^), and *m*/*z* 137.0939 ([M+H–C_7_H_17_O_7_]^+^) [35]. Dihydroconiferin has been described in stingless bee emerald honey from Bangkok (Thongsaiklaing et al., 2024) and in aroeira, betônica, coffee, cipó-uva, pequi, and multifloral velame honeys from Northern Minas Gerais [35]. It has also been found in plant leaves from various families [85,86,87] and has been associated with antifungal activity [86]. In Nicotiana benthamiana seeds, coniferin biosynthesis was linked to lignin regulation, fundamental for structural resistance and pathogen defense in plants [88].

Dehydrovomifoliol, molecular formula C_13_H_18_O_3_, was annotated from the major protonated ion [M+H]^+^ at *m*/*z* 223.1337, with characteristic fragments at *m*/*z* 205.1228 ([M+H–H_2_O]^+^) and *m*/*z* 121.0624 ([M+H–C_5_H_10_O_2_]^+^) [41,45]. Dehydrovomifoliol has been reported in sulla and dill honeys from Sicily [41], manuka honey from New Zealand [45], and in Greek monofloral honeys (fir, oak, pine, thyme) [69], as well as in betônica, aroeira, coffee, cipó-uva, pequi, velame, and multifloral honeys from Northern Minas Gerais [35]. It is a sesquiterpenoid [89], found in high concentrations in certain manuka honeys and used as a chemical marker for botanical origin [45]. Dehydrovomifoliol exhibits phytotoxic activity, inhibiting weed growth [90], potential for non-alcoholic fatty liver disease treatment [91], and cytotoxic activity against human cancer cell lines including HONE-1, KB, and HT29 [89].

Vomifoliol, molecular formula C_13_H_20_O_3_, was annotated from the major protonated ion [M+H]^+^ at *m*/*z* 225.1483, with characteristic fragments at *m*/*z* 207.1387 ([M+H–H_2_O]^+^), *m*/*z* 189.1270 ([M+H–2H_2_O]^+^), and *m*/*z* 149.0943 ([M+H–C_3_H_8_O_2_]^+^) [35]. It has been identified in *Mentha* spp. monofloral honeys [92] and in betônica, aroeira, cipó-uva, and pequi honeys [35]. Vomifoliol, an isoprenoid derivative [93,94], has been extensively reported for anti-inflammatory effects (via modulation of inflammation, inhibition of inflammatory enzymes, and reduction of pro-inflammatory cytokines) [93,95], moderate antioxidant activity [96], biocompatibility (low toxicity and high cellular compatibility in human and animal cells) [97], therapeutic potential for neurodegenerative diseases [98], neuroprotective effects against Alzheimer’s progression [99], antidiabetic activity through α-amylase and α-glucosidase inhibition [94], moderate antibacterial [96], antifungal [100] and antileishmanial activities [98].

Jasmonoyl-L-isoleucine (JA-Ile), molecular formula C_18_H_29_NO_4_, was annotated from the major protonated ion [M+H]^+^ at *m*/*z* 324.2180, with characteristic fragments at *m*/*z* 306.2074 ([M+H–H_2_O]^+^), *m*/*z* 278.2121 ([M+H–CO_2_H]^+^), *m*/*z* 151.1111 ([M+H–C_8_H_14_NO_3_]^+^), and *m*/*z* 132.1003 ([M+H–C_12_H_18_O_2_]^+^) [35]. JA-Ile has been reported in pequi and cipó-uva honeys [35]. It is the main signaling phytohormone of the jasmonate class [97]. JA-Ile exerts dual protective functions in host–parasite interactions, favoring parasite–host coexistence [77]. Under abiotic stress, JA-Ile rapidly accumulates in wounded tissues, inducing secondary metabolism genes and stimulating defense compound production such as terpenoids, phenolics, and flavonoids [97]. In wheat, JA-Ile exhibited a late stress response under saline-alkali conditions, suggesting it is not the primary regulator but acts in later stages to reinforce defense and adaptation mechanisms [101]. In herbivory studies with rice and leafhoppers, JA-Ile signaling induced the accumulation of sakuranetin, an antifungal flavonoid that inhibits insect symbionts, compromising nutrition and reducing damage to plants [102].

### 3.6. Antibacterial Activity Assay

The World Health Organization published in 2022 a document highlighting the importance of using minimally invasive methods in dental treatment to prevent caries, as this helps prolong the natural longevity of teeth and prevents pain, infection, or permanent dental damage [103].

Honey also represents a promising natural alternative for the treatment of various dental conditions, due to its antioxidant, antimicrobial, anti-inflammatory, and wound-healing activities, with proven efficacy in reducing dental plaque, gingivitis, and oral ulcers [104].

The avocado monofloral honey analyzed in this study did not show the ability to inhibit the growth of any of the tested microorganisms. It should be noted that the honey was tested at a concentration of 20%, and higher concentrations could have revealed antibacterial activity. Therefore, the absence of antibacterial activity against *Streptococcus salivarius*, *S. mitis*, *S. sanguinis*, *S. mutans*, *S. sobrinus*, *Lactobacillus paracasei*, and *Enterococcus faecalis* under the conditions used in this study may be attributed to different factors, due to the low concentration of honey used, insufficient amounts of active compounds, or weak synergistic interactions among the present substances to elicit a biological response in the assay [105]. Moreover, it is possible that the honey sample did not contain metabolites with specific antibacterial activity against the investigated species [106,107].

Among the compounds identified in the avocado honey sample, some have been widely described in the literature as exhibiting antimicrobial activity. For example, scopoletin shows significant antibacterial activity and inhibits the growth of *Staphylococcus aureus*, *Enterococcus faecium*, *Stenotrophomonas maltophilia*, and *Pseudomonas aeruginosa*. Its reported mechanisms include bacterial cell wall lysis and deformation, resembling the action of some antibiotics [70,71]. The compound 8-hydroxyquinoline (an isomer of 2-hydroxyquinoline) and its derivatives display activity against a wide range of microorganisms, including *Mycobacterium tuberculosis*, *Escherichia coli*, *Staphylococcus aureus*, and *Streptococcus mutans*, partly due to their ability to form complexes with essential metal ions, thereby inhibiting bacterial growth and inducing cell death [63]. Vomifoliol, in turn, has been reported to exert moderate antibacterial activity against *Staphylococcus aureus*, possibly associated with the induction of oxidative stress [96].

However, it is plausible that, in the analyzed honey, the concentrations of these compounds were far below the levels required to exert an inhibitory effect against the tested bacteria. In addition, intrinsic characteristics of the honey matrix, such as high viscosity, sugar-rich composition, potential chemical interactions among constituents, and the hydrophilic nature of some phenolic compounds, may limit their interaction with the bacterial membrane.

The results observed in this study are consistent with those reported in honey samples from different regions of Minas Gerais, where monofloral honeys of *Stachytarpheta cayennensis* (betônica), *Cissus verticillata* (cipó-uva), *Myracrodruon urundeuva* (aroeira), *Caryocar brasiliense* (pequi), and *Croton* sp. (velame) did not show activity against *S. faecalis*, *S. mitis*, *L. paracasei*, and *S. mutans*. Against *S. sanguinis*, these honeys presented MIC values ranging between 10% and 20%. Only aroeira and pequi honeys inhibited *S. salivarius*, pequi honey was the only one effective against *S. sobrinus*, and multifloral honey was the only sample that inhibited all these strains, with both MIC and MBC values [12]. South African honeys showed low antibacterial activity, with MIC values of 25% against *S. salivarius*, *S. sanguinis*, *S. mutans*, and *S. sobrinus*, except for wild honey, which presented a MIC of 50% against *S. salivarius* [108]. Some studies also demonstrated antibacterial inhibition of *S. mutans* by honeys from various geographical and botanical origins, with MIC values ranging from 6–25% [109], and MIC of 5% [110]. In the latter, involving *Nigella sativa* honey, the combination of *N. sativa* honey with ethanolic seed extract exhibited a significant inhibitory effect [110].

The antibacterial activity of honey may be enhanced by the presence of polyphenols, particularly flavonoids [12]. However, they may be present only at very low concentrations, likely insufficient for their combined action to have played a relevant role in inhibiting bacterial growth as reported in previous studies.

## 4. Materials and Methods

### 4.1. Chemicals and Instruments

All reagents and chemicals used were analytical grade from Sigma Chemical Company (St. Louis, MO, USA). The solvents and reagents used in the experiments included methanol, acetonitrile, ethyl acetate, sodium chloride, anhydrous sodium sulfate, glycerin, paraffin, Folin–Ciocalteu reagent, sodium carbonate, gallic acid, 2,2-diphenyl-1-picrylhydrazyl (DPPH), and resazurin. Methanol, acetonitrile, and formic acid used for LC-MS/MS analysis were of MS grade (≥99.9% purity) and obtained from Sigma-Aldrich (St. Louis, MO, USA). Ultrapure water was produced using a Milli-Q system (Millipore, Bedford, MA, USA). A spectrophotometer (Shimadzu UV-VIS 2550/Tokyo, Japan) was used for absorbance measurements and a refractometer (VODEX VX090/Vitória, ES, Brazil) for moisture determination. The chromatograms were obtained using a UHPLC system (Shimadzu, Kyoto, Japan) consisting of a chromatograph with a Nexera LC-30AD pump (Shimadzu, Kyoto, Japan), connected to an autosampler Nexera SIL-30AC (Shimadzu, Kyoto, Japan) and a DAD Nexera SPD-M20A detector (Shimadzu, Kyoto, Japan), supervised by a CBM Nexera 20 A (Shimadzu, Kyoto, Japan). The chromatograph was coupled to a mass spectrometer with time-of-flight detection model maXis-ETD ESI-QqTOF (Bruker, Karlsruhe, Germany). Separations were performed on a Shimpack XR-ODSIII, C18, 2.2 µm, 80 A, 2.0 × 150 mm column (Shimadzu, Kyoto, Japan).

### 4.2. Honey Samples

The avocado honey (*Apis mellifera*) samples were collected at the Tsuge Group farm, located in the municipality of São Gotardo, MG, taken to the Cooperative of Family Beekeepers of Northern Minas Gerais (COOPEMAPI, based in Bocaiuva, MG, Brazil), and provided for analysis, samples were received by the cooperative in (August to October) 2022. The samples were identified by numbering and stored protected from light (25 to 30 °C). All methods are based on specialized literature, including the Codex Alimentarius, Association of Official Analytical Chemists (AOAC), Normative Instruction number 11 of 10/20/2000 [34,111,112] and publications of the International Honey Commission. Experiments were performed in triplicate and all results are shown as mean +/− SD. The study was registered in SISGEN under the number AA83FF6.

### 4.3. Botanical Identification

The microscopic slides were prepared by dissolving 10 g of honey in 20 mL of deionized water. After centrifugation, the pellet was embedded in unstained glycerin gelatin and the slides sealed with paraffin. The amount of pollen of the species was observed and the result was interpreted by the dominance of pollen thiop. The pollen count analysis was performed as described by Barth, 2004 and Louveuax et al., 1978 [30,33], using the database provided by PROBEE Ltd. (Plzeň, Czech Republic).

#### Determination of the Honey Color

This analysis was performed according to the methodology proposed by the Codex Alimentarius Commission [34], which consists of reading the absorbance of the pure sample in a spectrophotometer at 560 nm against pure glycerin blank. Classification was performed according to the Pfund table.

### 4.4. Total Polyphenols

To determine total phenolics, the Folin–Ciocalteu method was used with modifications [113]. For the assay, natural (unprocessed) honey solubilized in water, in concentrations ranging from 20 to 120 mg mL^−1^ (250 μL), was added to 2.75 mL of 3% Folin–Ciocalteu reagent. After 5 min, 250 μL of 10% sodium carbonate was added. The solution was kept protected from light for one hour at 25 °C. Absorbance was measured at 765 nm, using a blank of water (250 μL), 3% Folin–Ciocalteu (2.75 mL) and 10% sodium carbonate, with a UV-VIS spectrophotometer (SHIMADZU-UV-VIS 2550, Shimadzu, Duisburg, Germany). All measurements were performed in triplicate, and the results were calculated and plotted on a concentration/absorbance graph to determine the equation of the line and R^2^. Gallic acid (20–140 μg mL^−1^) was used as a standard to derive the calibration curve. Total phenolic content was expressed as mg of gallic acid equivalent per 100 g of honey. The experiment was performed in triplicate and all results are shown as mean ± SD.

### 4.5. Antiradical Capacity

The free radical scavenging activity of 2,2-diphenyl-1-picrylhydrazyl (DPPH) in the honey samples was determined as described by Brand-Williams et al. (1995) [114], with the adaptations described below. The samples were prepared by dissolving 2 g of honey in 100 mL of methanol (stock solution: 20 mg·mL^−1^). For the assay, aliquots of 500 µL of the sample were used at concentrations ranging between 2 and 14 mg·mL^−1^. A stock solution of DPPH was prepared in methanol at a concentration of 40 µg mL^−1^, from which 3000 µL were taken and added to the sample, then shaken vigorously and kept in the dark for 25 min. at 25 °C. For the preparation of the gallic acid calibration curve, a stock solution of 80 µg mL^−1^ was prepared, from which working standards ranging from 0.5 to 3.5 µg mL^−1^ were obtained. The absorbance of each solution was measured at 517 nm using a UV–Vis spectrophotometer (SHIMADZU UV-2550, Tokyo, Japan) against a methanol blank. All measurements were taken in triplicate and the results are reported as mean ± SD. With the absorbance values, the percentage of antioxidant activity was calculated by the equation [115]:

{(A_control_ − A_sample_)/A_control_} × 100,

where A_control_ represents the absorbance value of the control; A_sample_ represents the absorbance value of the sample.

The EC_50_ value was determined using the equation of the line obtained from the absorbance readings across the tested concentration range.

### 4.6. Honey Extraction

The sample (5 g) was transferred to a pre-tared scintillation vial. Due to the viscosity of the sample, it was necessary to heat it in a water bath at 45 °C. An amount of 50 mL of NaCl solution (2% *w*/*v*) was prepared. In this way, 1 g of NaCl was dissolved in 50 mL of ultra-pure water. An amount of 5 mL of this solution was added to the sample. This material was vortexed for 10 s. Then extraction was performed with ethyl acetate (5× with 5 mL). The organic phase was separated and Na_2_SO_4_ was added thereto, stirred, filtered through cotton into a pre-tared scintillation vial. The solution was dried in speedvac producing the ethyl acetate fraction [116].

### 4.7. Preparation of Solutions for Analysis

The previously obtained ethyl acetate partition was solubilized in a methanol/water solution (3:2, *v*/*v*), resulting in a final concentration of 5 µg/µL. The solution was then subjected to ultrasonication for 10 min and subsequently centrifuged at 10,000 rpm for 15 min. After this process, the sample was kept at room temperature for approximately 24 h.

The sample solution was transferred to the UHPLC autosampler vial. For extraction process control, a 1000 µL methanol/water solution was prepared. As reference substances, the following standards were used: gallic acid, caffeine, quercetin and rutin. Standard solutions were diluted in a 3:2 (*v*/*v*) methanol/water solution, according to their respective molar masses, obtaining solutions at a concentration of 5 µg/µL [116].

### 4.8. Analysis Method LC-MS/MS

The mobile phase consisted of (A) water with 0.1% formic acid and (B) acetonitrile with 0.1% formic acid. A linear gradient from 5 to 95% B in 15 min was used. Between each injection of 5 µL of each sample, the column was reconditioned with 95% B for 3 min and 5% B for 6 min. The spectrometer operated as follows: Ion source type: ESI; positive polarity; scan 100 at 1500 *m*/*z*; nebulizer gas: 3.0 bar; drying gas flow: 8 L/min; temperature: 200 °C [116].

### 4.9. Antibacterial Activity Assay

#### 4.9.1. Cariogenic Bacteria

The bacteria used in this study were obtained from the American Type Culture Collection (ATCC): *Streptococcus salivarius* (ATCC 25975), *S. mitis* (ATCC 49456), *S. sanguinis* (ATCC 10556), *S. mutans* (ATCC 25175), *S. sobrinus* (ATCC 33478), *Lactobacillus paracasei* (ATCC 11578), *Enterococcus faecalis* (ATCC 4082). All the bacteria were kept in the Laboratory of Antimicrobial Assays (LEA) of the Federal University of Uberlândia, Brazil at −20 °C, in 80% glycerol solution.

#### 4.9.2. Minimum Inhibitory Concentration and Minimum Bactericidal Concentration

Minimum inhibitory concentration (MIC) is defined as the lowest concentration of an extract, fraction or compound that can inhibit bacterial growth. The experiment was performed in 96-well microplates and repeated three times.

For the assays, stock solutions were prepared prior to each batch of testing at concentrations up to 20% (*w*/*v*) natural (unprocessed) honey in Brain Heart Infusion broth (Difco, Detroit, MI, USA). Solutions were vortexed until completely dissolved, then sterilized by serial filtration through 0.22 μm polyethersulfone (PES) membranes (Millipore, Tullagreen, Carrigtwohill, Country Cork, Ireland) to eliminate contaminating spore-forming organisms. The tested concentrations of the samples ranged from (% *w*/*v*) 0.009%, 0.019%, 0.039%, 0.078%, 0.15%, 0.3%, 0.6%, 1.25%, 2.5%, 5%, 10% and 20%, the control (chlorhexidine) was tested at concentrations between 0.000012% to 0.0059%, and the inoculi were adjusted to a cell concentration of 5 × 10^5^ CFU mL^−1^ [117]. Inoculated wells containing bacteria were only included to control growth. Noninoculated wells (without any bacteria) were also employed to ensure broth sterility. The 96-well microplates were incubated at 37 °C for 24 h. After incubation, 30 μL of 0.02% aqueous resazurin solution was added to each well to observe microbial growth. The blue and red colors represent the absence and presence of microbial growth, respectively [118].

MBC is defined as the lowest concentration of the sample where no bacterial growth occurs. A substance is considered to exert a bacteriostatic effect when its MBC value is higher than its MIC value. However, a substance is considered to exhibit a bactericidal effect when its MBC value is the same as its MIC value. To determine MBC, 10 μL of the inoculum, removed from each well before resazurin was added, was plated on blood agar supplemented with 5% defibrinated horse blood. The plates were incubated in a bacteriological oven or anaerobiosis chamber at 37 °C for 24 h [119].

## 5. Conclusions

The present study demonstrated that the analyzed avocado honey can be classified as monofloral, with a high content of phenolic compounds and strong antioxidant activity. Chemical characterization by LC-MS/MS revealed the presence of relevant bioactive metabolites, including scopoletin, caffeine, abscisic acid, and vomifoliol, reinforcing the functional potential of this honey. Although no significant antibacterial activity was observed against the tested oral strains, this may be attributed to factors such as the concentrations used, the specific bacterial species, or the hydrophilic nature of the phenolic compounds. Nonetheless, the rich phenolic composition and metabolic diversity support the nutritional and therapeutic value of avocado honey. These findings suggest that further studies are warranted to explore other biological effects. Overall, this study expands current knowledge on the bioactive properties of Brazilian monofloral honeys and highlights avocado honey as a promising natural resource with potential applications in human health and the development of innovative products.

## Figures and Tables

**Table 1:** Botanical origin—percentage index of apicultural species presents in avocado honey (*Persea americana* Mill.).

Pollen Type	Numerical Counting of Pollen Grains	Percentage Index (%)
Family—Lauraceae Species—*Persea americana* Mill	200	86.21
Family—Myrtaceae Species—*Eucalyptus* sp.	15	6.46
Family—Sapindaceae Species—*Serjania lethalis*	7	3.02
Family—Anacardiaceae Species—*Astronuim urundeuva*	5	2.15
Family—Compositae Species—*Baccharis* sp.	5	2.15
Total	232	100%

**Table 2:** Compounds annotated by UHPLC-ESI-MS/MS in avocado honey and compared with literature data.

Peak Id.	Compound Annotated	RT (min)	MS [M+H]^+^ (*m*/*z*)	Mass Error (ppm)	MS/MS Fragments (*m*/*z*)	Molecular Formula	Literature Reference
20	2-Hydroxyquinoline ^a,c^	6.2	146.0589	7.4	117.0552; 118.0620	C_9_H_7_NO	[35]
29	Caffeine ^a,b,c^	10.4	195.0879	−1.3	138.0648; 110.0720	C_8_H_10_N_4_O_2_	[35,36]
38	Scopoletin ^a,b,c^	11.6	193.0497	−1.5	137.0581; 165.0545; 177.0549	C_10_H_8_O_4_	[37,38]
39	Abscisic acid ^c^	11.8	265.1441	−2.6	201.1284; 229.1222; 247.1334	C_15_H_20_O_4_	[39,40,41,42]
81	Abscisic acid isomer ^c^	16.4	265.1441	−3.6	153.0906; 229.1222; 247.1334		
43	Tuberonic acid ^c^	12.5	227.1286	−3.5	131.0839; 149.0950; 163.1110; 191.1069; 209.1179	C_12_H_18_O_4_	[35,43,44]
56	Dehydrovomifoliol ^c^	14.0	223.1337	−3.6	121.0624; 205.1228	C_13_H_18_O_3_	[41,45]
66	Dihydroconiferin ^c^	14.9	345.1530	4.0	137.0939; 165.0913; 183.1019	C_16_H_24_O_8_	[35]
73	Vomifoliol ^c^	15.6	225.1483	1.0	149.0943; 189.1270; 207.1387	C_13_H_20_O_3_	[35]
144	Jasmonoyl-L-isoleucine ^c^	22.9	324.2180	−3.2	132.1003; 151.1111; 260.2022; 278.2121; 306.2074	C_18_H_29_NO_4_	[35]

^a^ Mass-Bank, precursor ion [M+H]^+^ and product ion mass spectrum matched with those recorded at “Mass-Bank” MS/MS library; ^b^ In-house, precursor ion [M+H]^+^ and product ion mass spectrum matched with those recorded at “In-house” MS/MS library. ^c^ Target list, precursor ion [M+H]^+^ mass spectrum matched with those recorded on a list of targets specific to the plant’s family, genus and species. Id.—identification.

**Table 3:** Results of minimum inhibitory concentration/minimum bactericidal concentration (MIC/MBC).

Results in % (MIC/MBC)				
Caries Bacteria				
	Avocado Honey		Chlorhexidine (Control)	
	MIC	MBC	MIC	MBC
*Streptococcus mutans* (ATCC 25175)	>20	>20	0.000046	0.000046
*Streptococcus sobrinus* (ATCC 33478)	>20	>20	0.000092	0.000092
*Streptococcus mítis* (ATCC 49456)	>20	>20	0.00018	0.00018
*Streptococcus paracasei* (ATCC 11578)	>20	>20	0.00018	0.00018
*Streptococcus salivarius* (ATCC 25975)	>20	>20	0.00018	0.00018
*Streptococcus faecalis* (ATCC 4082)	>20	>20	0.00018	0.00018
*Streptococcus sanguinis* (ATCC 10556)	>20	>20	0.000092	0.000092

Concentrations of the samples tested against aerobic bacteria = 0.0098% to 20%.

## Data Availability

All data are published in the article.

## Supplementary Materials

The following supporting information can be downloaded at https://www.mdpi.com/article/10.3390/plants14213340/s1: Figure S1. UHPLC-ESI-QTOF-MS/MS profile of the avocado honey. Chromatograms registered in positive ionization mode (ESI+) showing all compounds detected from 0 to 60 min (A) and the substances annotaded from 5.0 to 24.0 min (B). Annotated compounds: see Table 2. Chromatographic and spectrometric conditions: see the materials and methods section. Below Figure S1, the mass spectra of the substances.

## Abbreviations

pH	Hydrogen potential
HPLC	High-Performance Liquid Chromatography
COOPEMAPI	Cooperative of Beekeepers and Family Farmers of Northern Minas Gerais
SISGEN	National System for the Management of Genetic Heritage and Associated Traditional Knowledgeinear
g	grams
mL	milliliters
ATCC	American Type Culture Collection
PES	Polyethersulfone
CFU	Colony Forming Unit
MIC	Minimum Inhibitory Concentration
MBC	Minimum Bactericidal Concentration
NaCl	Sodium Chloride
Na_2_SO_4_	Sodium Sulfate
rpm	Revolutions per Minute
ESI	Electrospray Ionization
*m*/*z*	mass-to-charge ratio
LC-MS/MS	Liquid Chromatography coupled to Tandem Mass Spectrometry
mg	miligram
GAE	Gallic Acid Equivalents
DPPH	2,2-diphenyl-1-picrylhydrazyl
PROBEE	Brazilian Program for Research and Development in Apiculture and Meliponiculture
Ltd	Limited liability company
EC_50_	Half Maximal Effective Concentration
RT	Retention Time
HONE-1	Human nasopharyngeal carcinoma cell line
KB	Human oral carcinoma cell line
HT29	Human colorectal adenocarcinoma cell line
AOAC	Association of Official Analytical Chemists
SD	Standard Deviation
nm	nanometer
uL	microliter
min.	minute
h	hour
TNF-α	Tumor Necrosis Factor alpha
IL-1β	Interleukin 1 beta
IL-12	Interleukin 12
IL-6	Interleukin 6
PGE2	Prostaglandin E2
COX-2	Cyclooxygenase-2
iNOS	inducible Nitric Oxide Synthase
ETD ESI-QqTOF	Electron Transfer Dissociation Electrospray Ionization Quadrupole Quadrupole Time-of-Flight
UHPLC-MS/MS	Ultra-High Performance Liquid Chromatography coupled with Tandem Mass Spectrometry
