# Supplementary material for: Compound Annotation by UHPLC-MS/MS, Quantification of Phenolic Compounds and Antimicrobial Activity of Monofloral Avocado Honey
Source: Plants (Basel). 2025 Oct 31;14(21):3340. doi: 10.3390/plants14213340 (PMC12608498; doi:10.3390/plants14213340)

**Suppementary Materials**

**Figure S1:** UHPLC-ESI-QTOF-MS/MS profile of the avocado honey. Chromatograms registered in positive ionization mode (ESI+) showing all compounds detected from 0 to 60 min (A) and the substances annotaded from 5.0 to 24.0 min (B). Annotated compounds: see Table 2. Chromatographic and spectrometric conditions: see the materials and methods section.

**(A)**

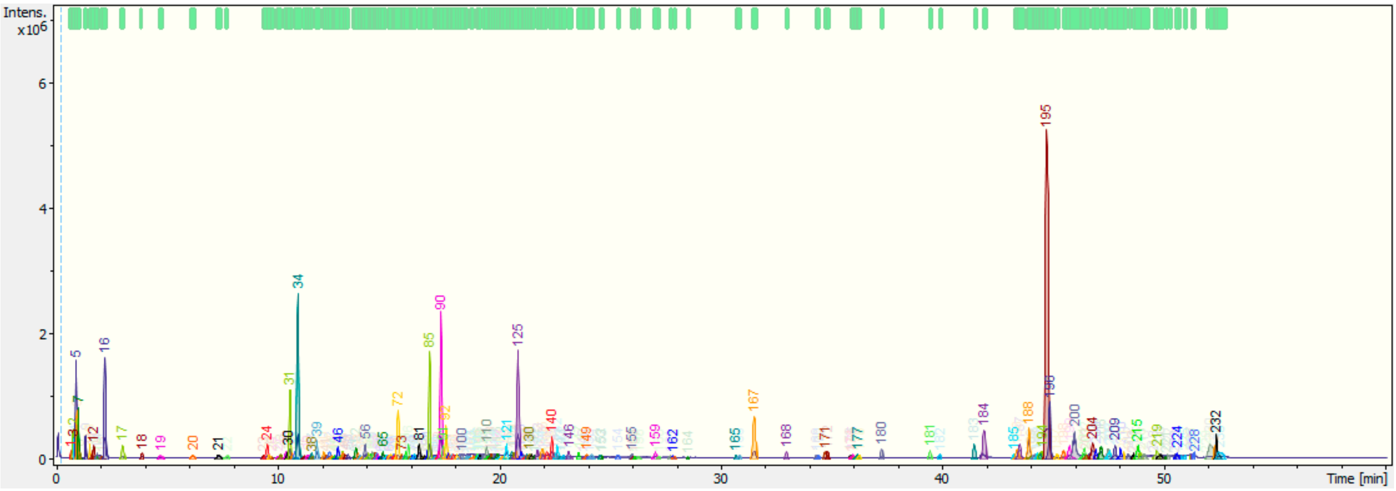


**(B)**

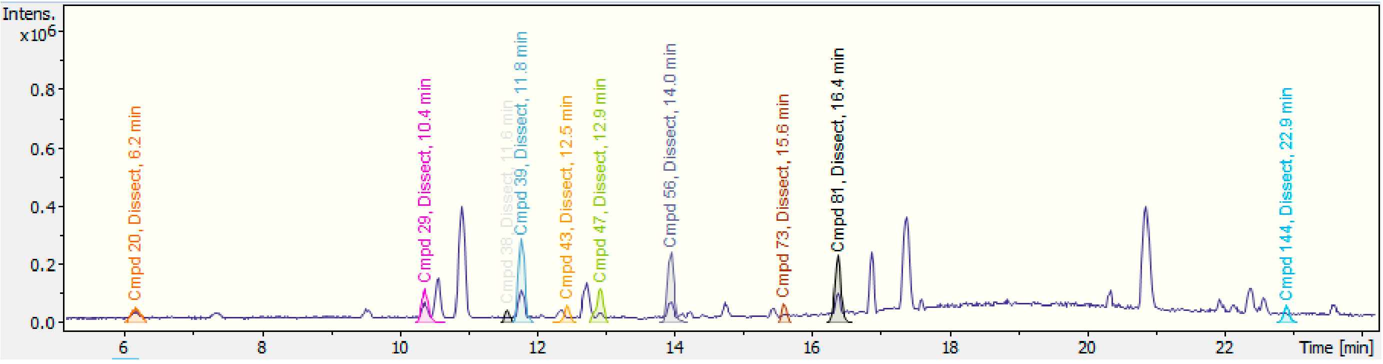


Spectra of the Compounds in Table 2

**Cmpd 20, Dissect, 6.2 min**

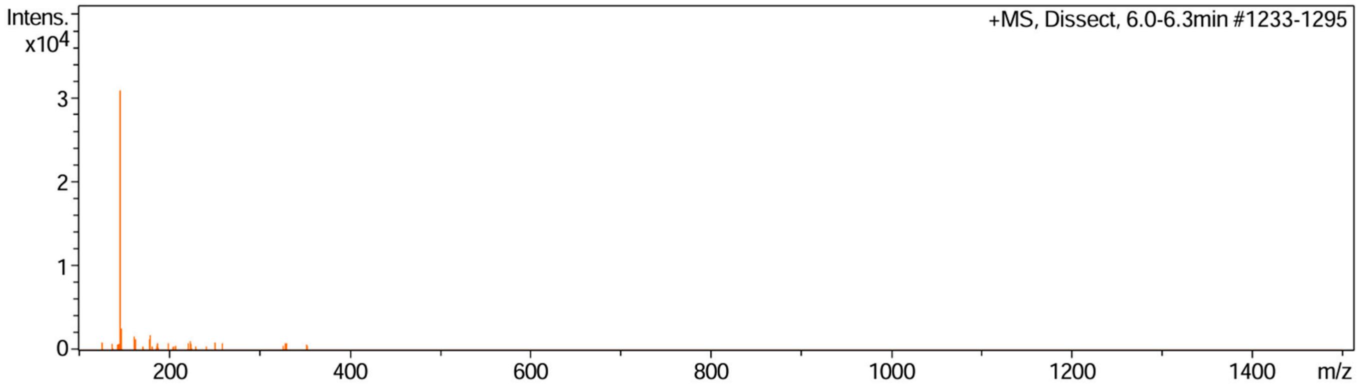

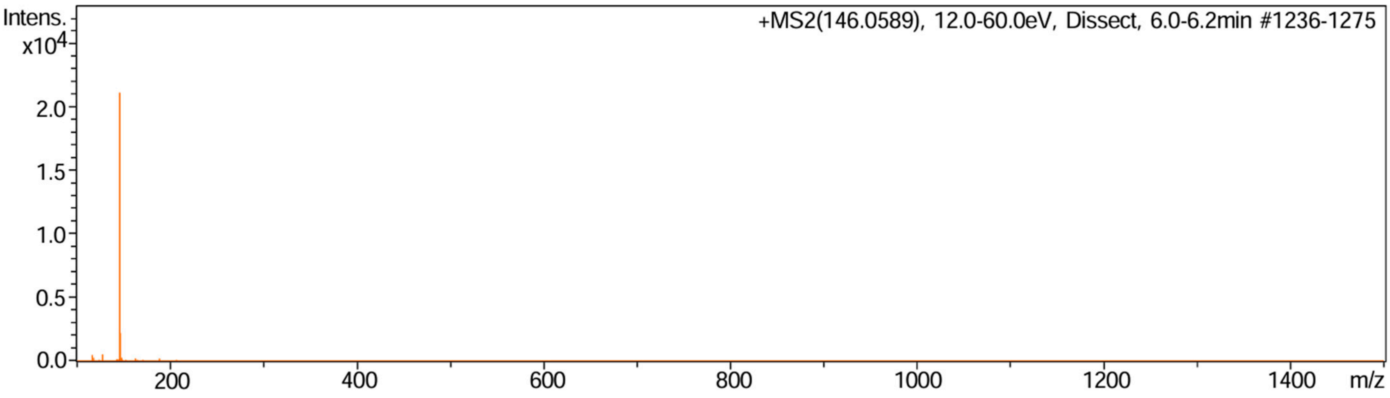


**Cmpd 29, Dissect, 10.4 min**

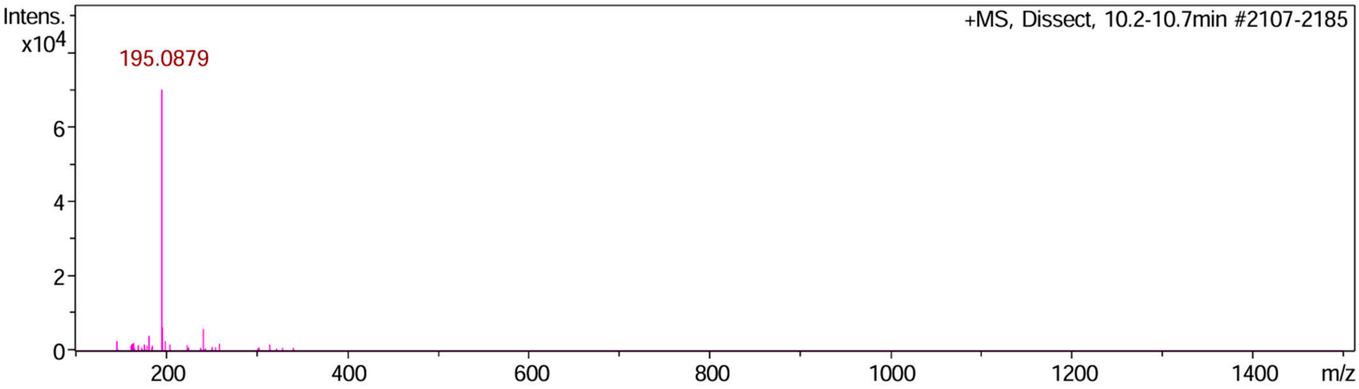


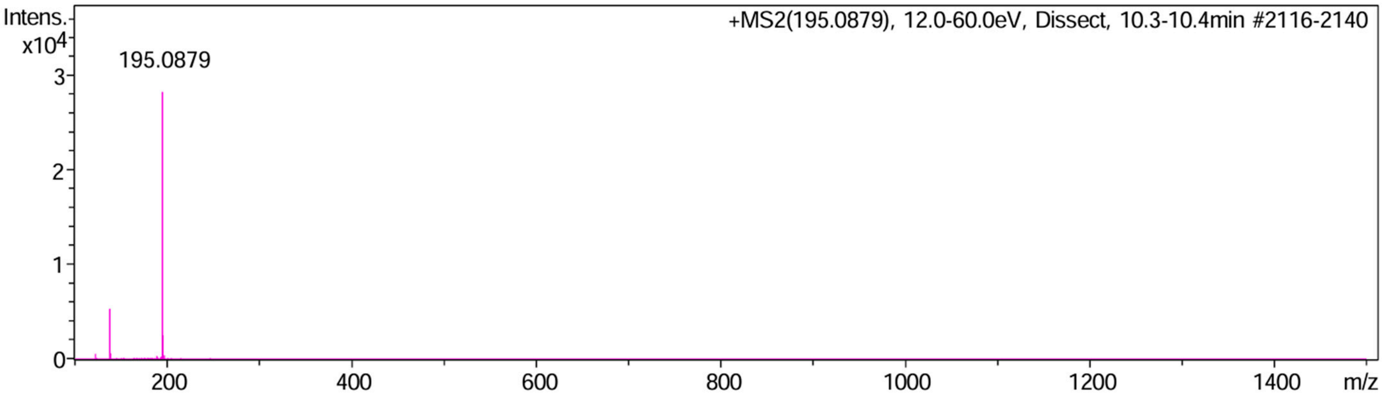


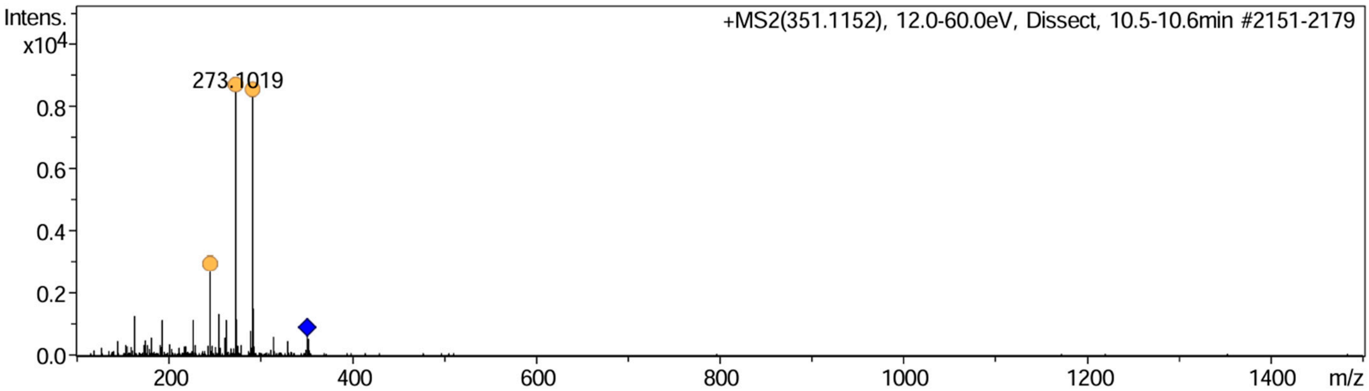

**Cmpd 38, Dissect, 11.6 min**

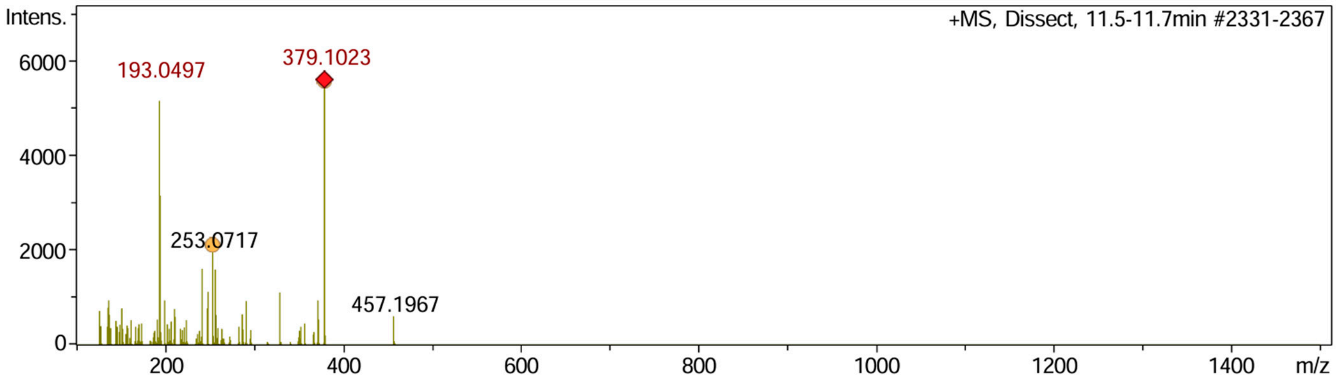


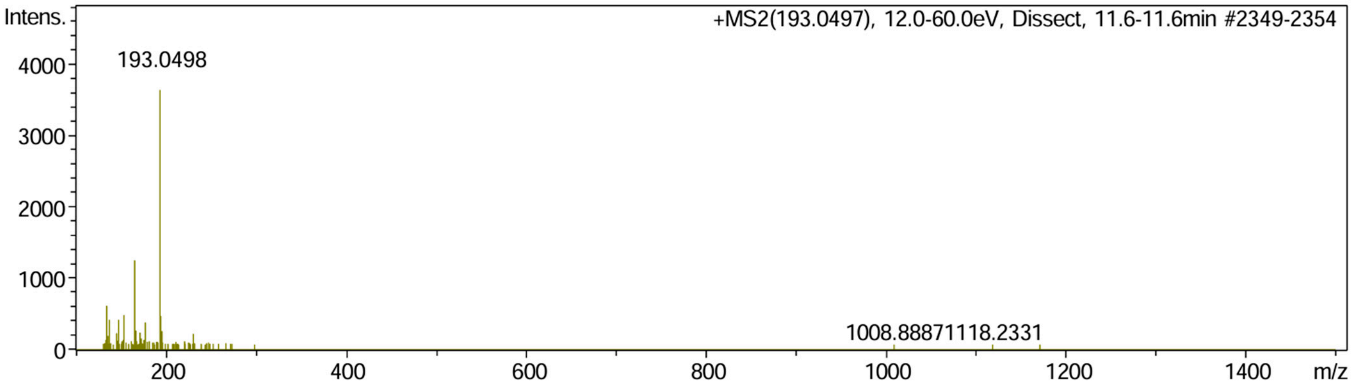


**Cmpd 39, Dissect, 11.8 min**

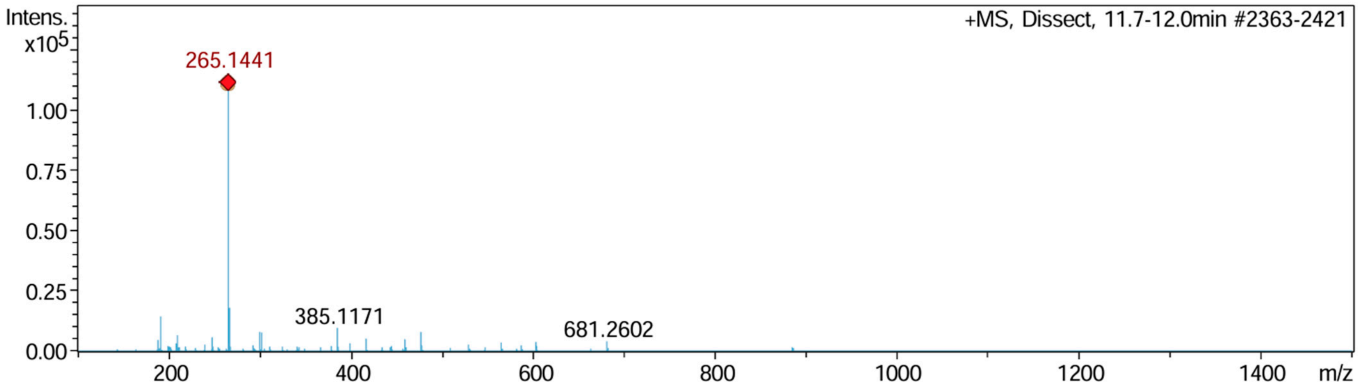


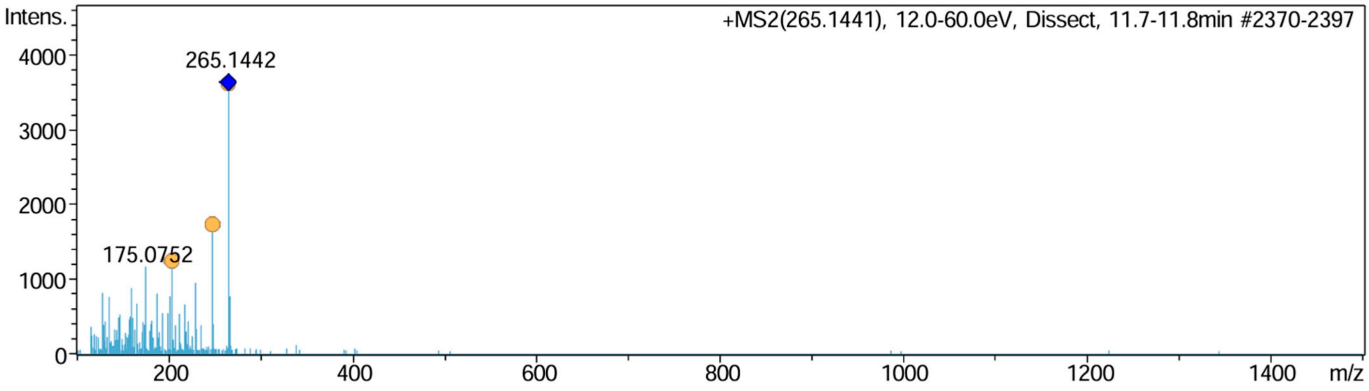

**Cmpd 43, Dissect, 12.5 min**

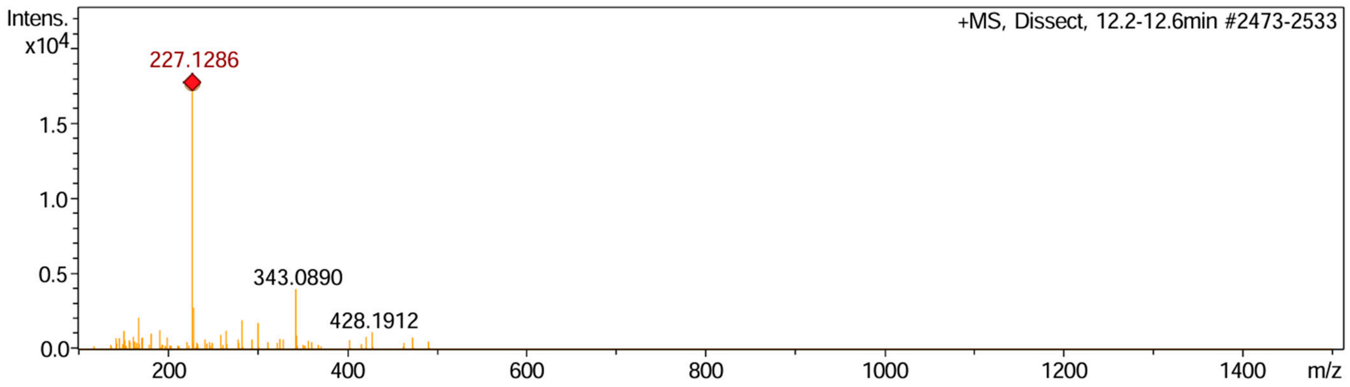


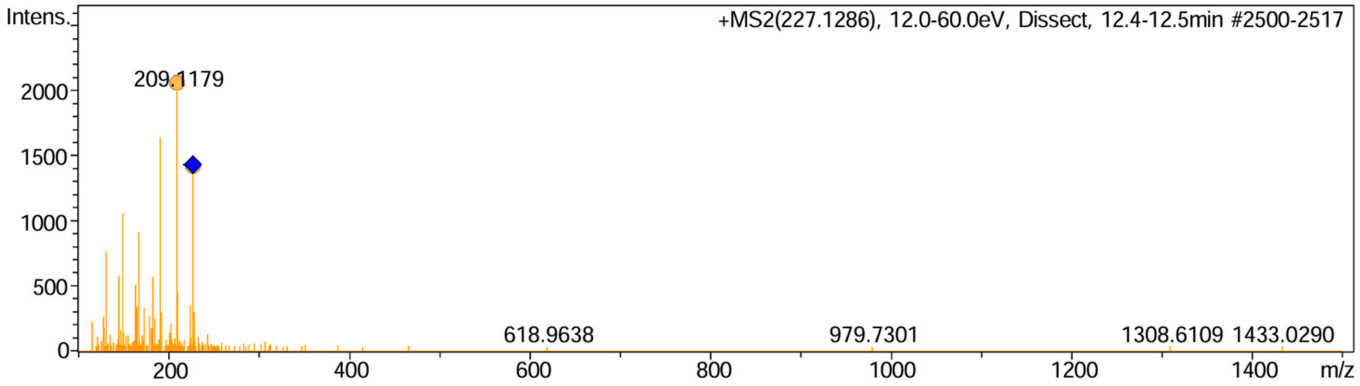


**Cmpd 56, Dissect, 14.0 min**

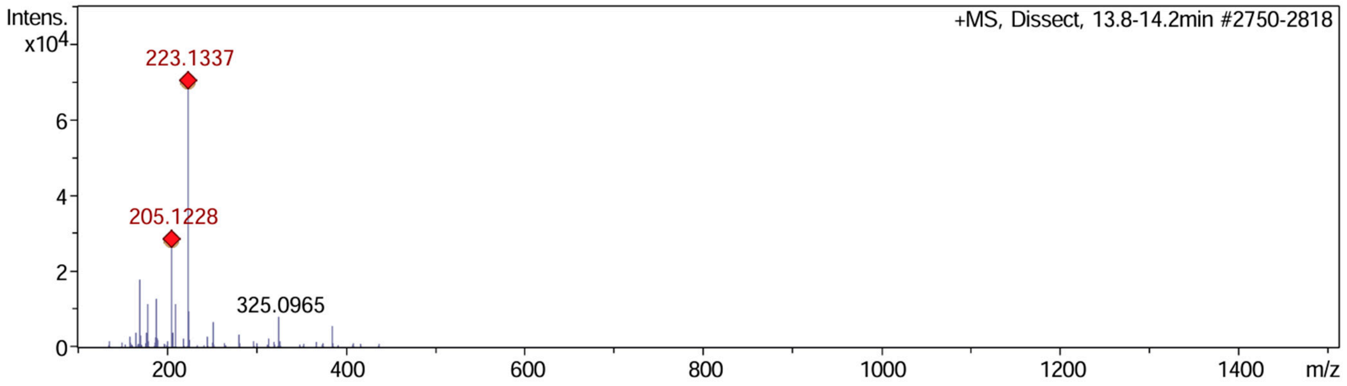

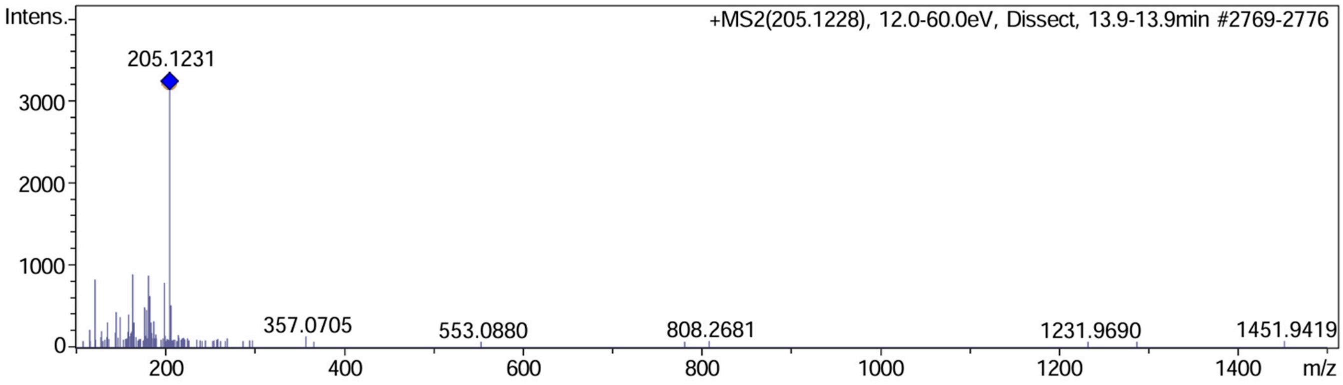


**Cmpd 66, Dissect, 14.9 min**

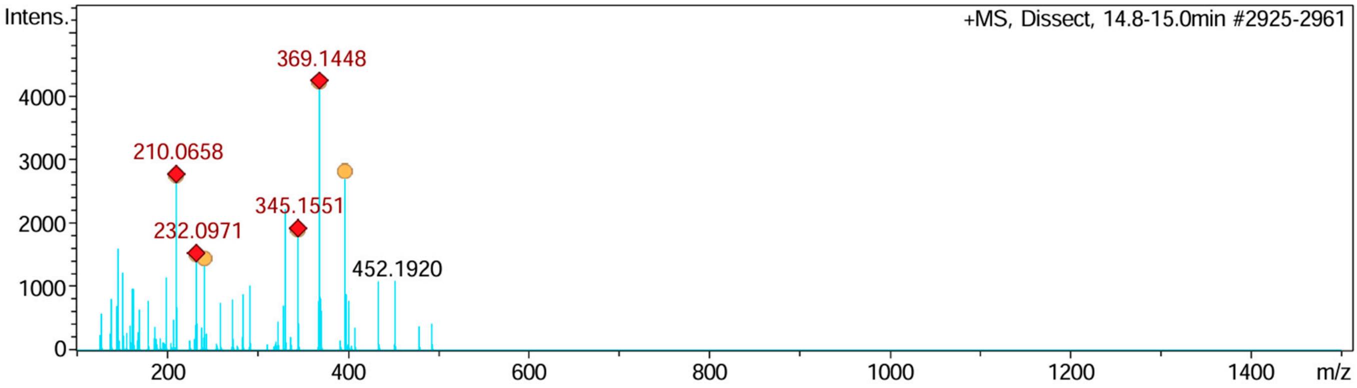


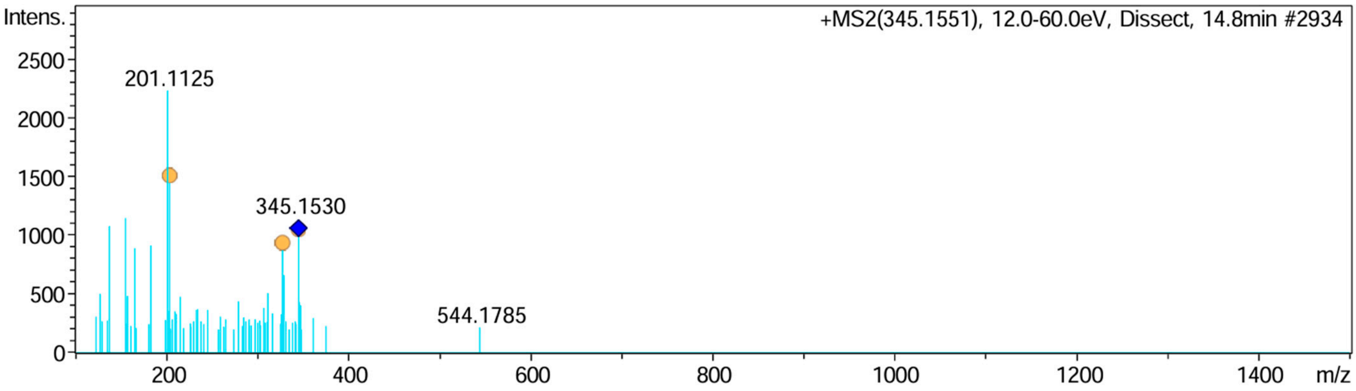

**Cmpd 73, Dissect, 15.6 min**

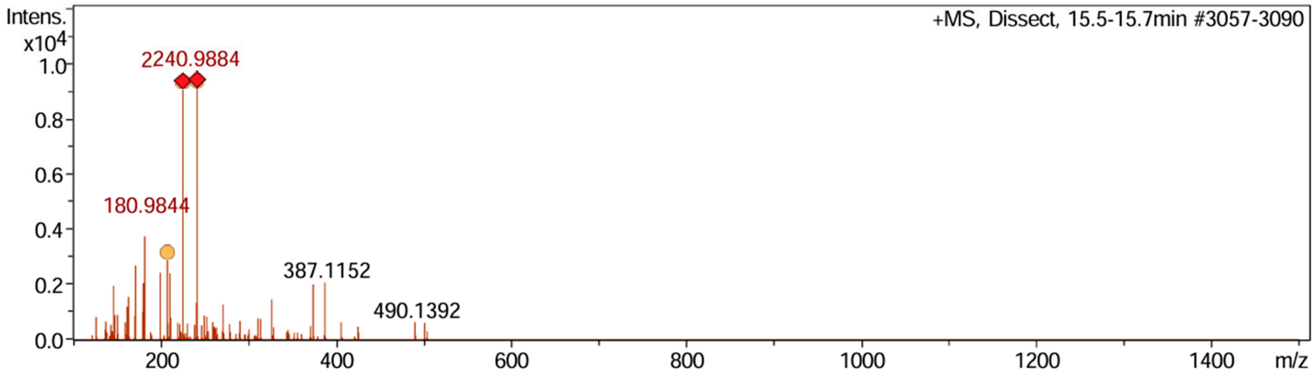


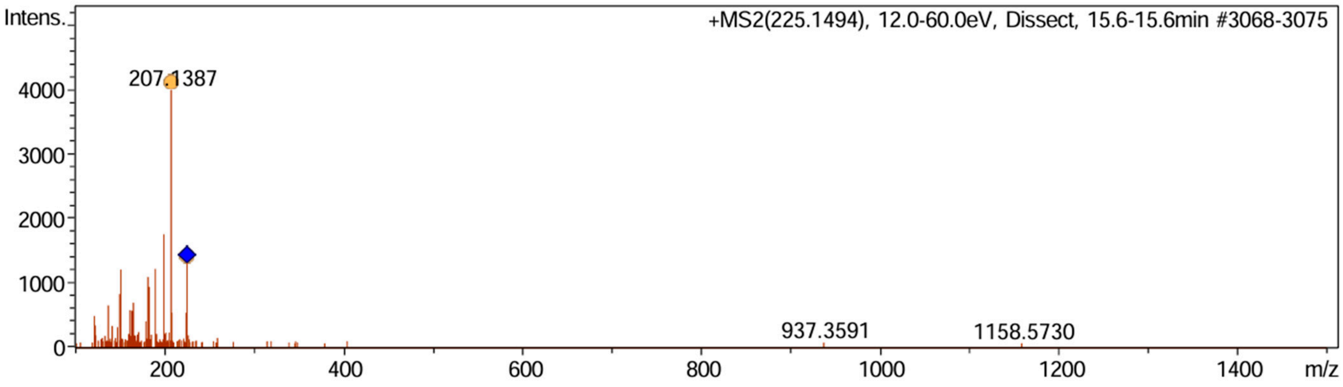


**Cmpd 81, Dissect, 16.4 min**

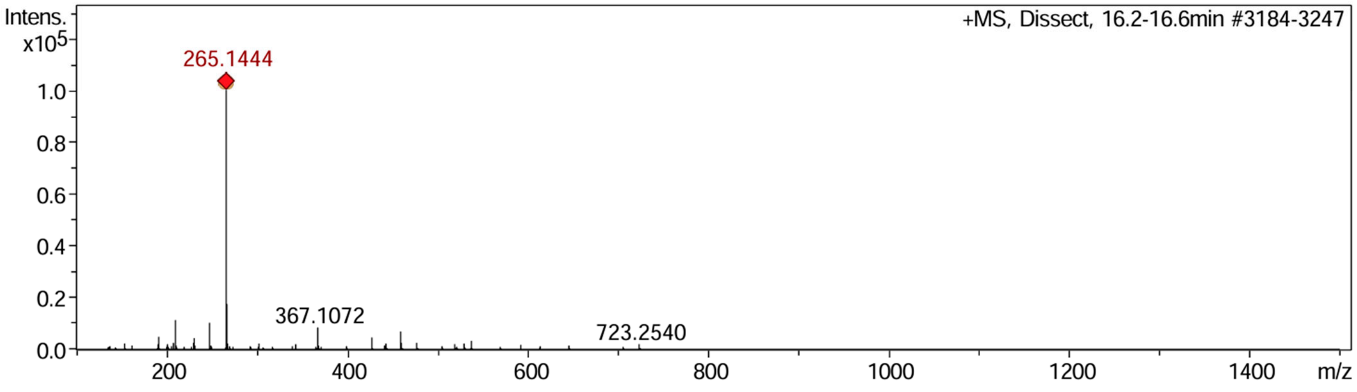

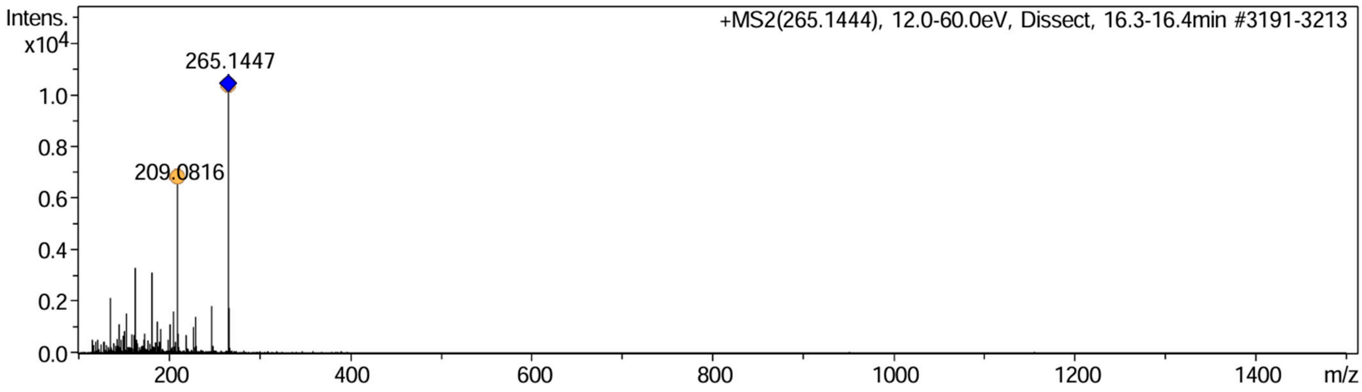


**Cmpd 144, Dissect, 22.9 min**

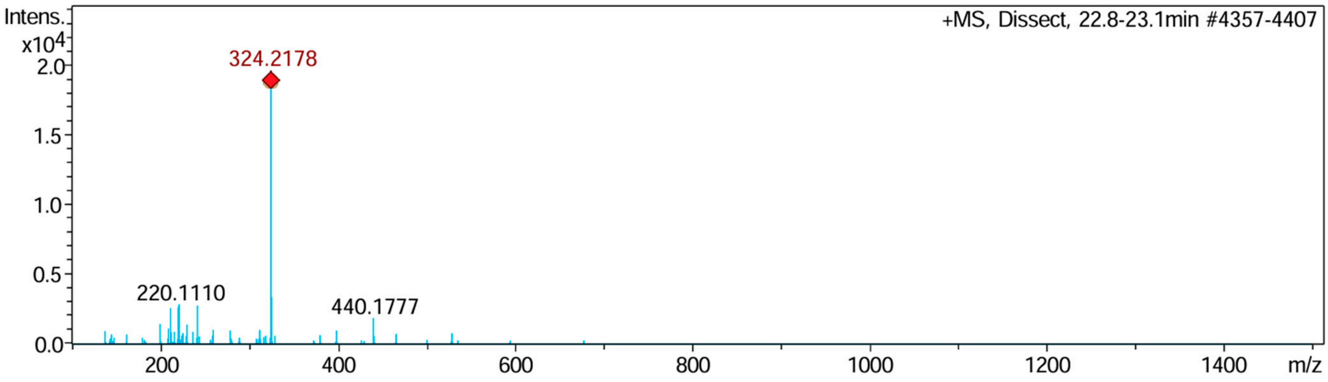


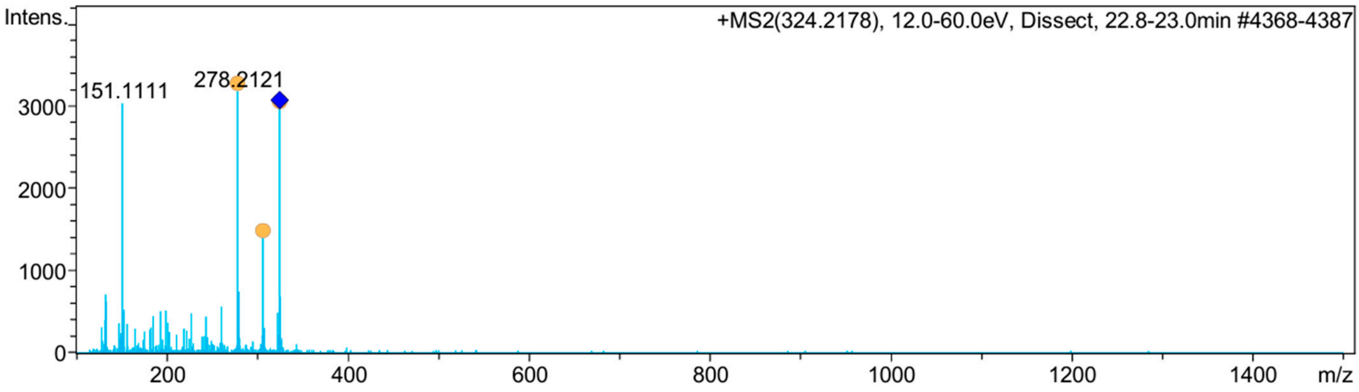

Spectrum of the unassigned peaks

**Cmpd 5, Dissect, 0.9 min**

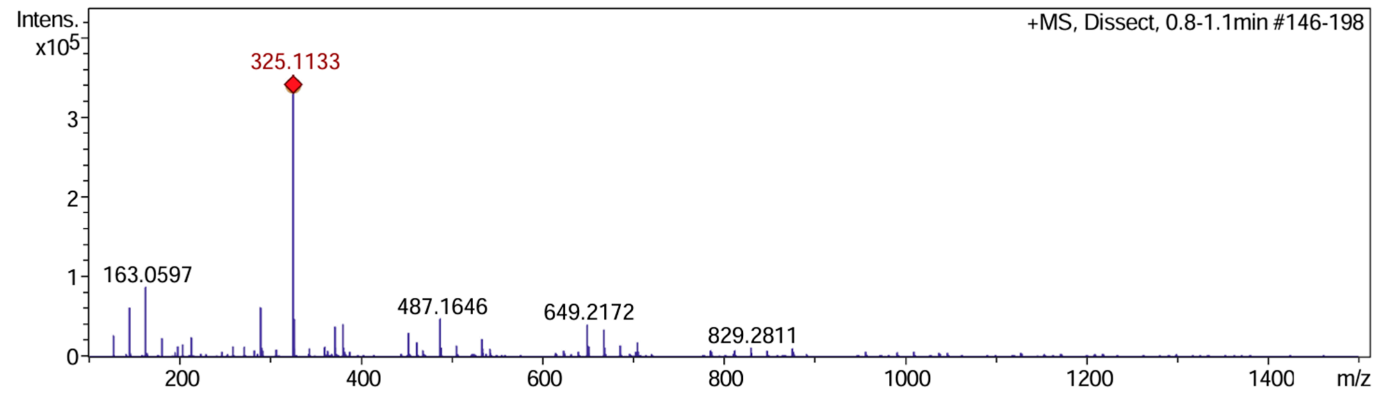


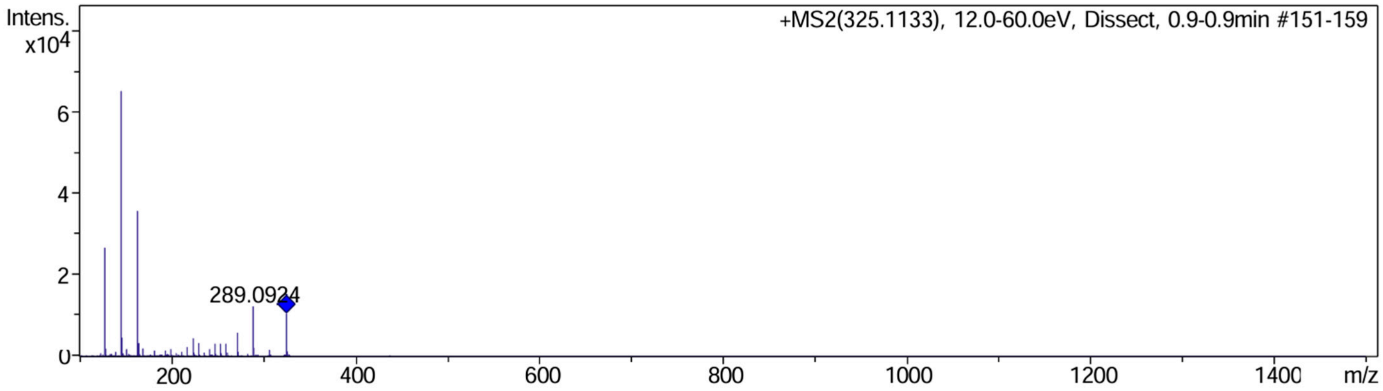


**Cmpd 16, Dissect, 2.2 min**

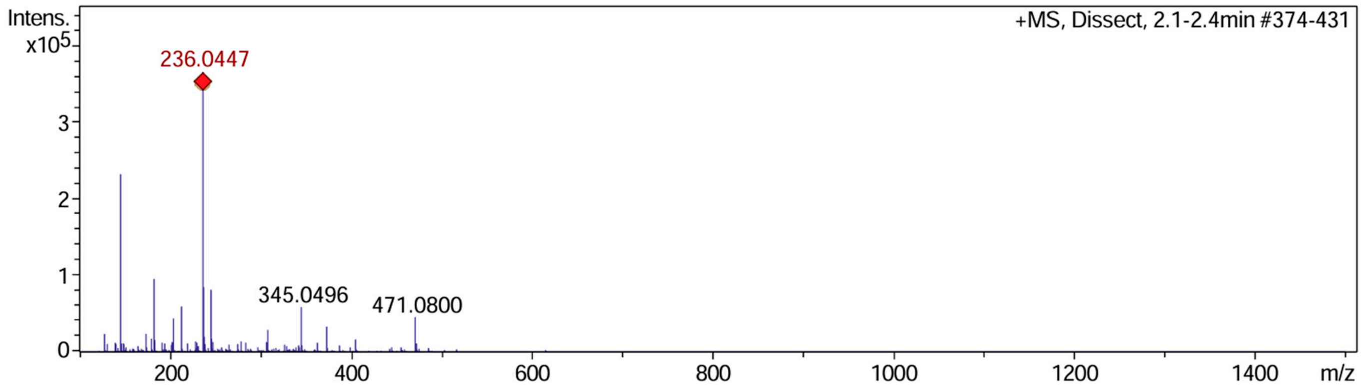

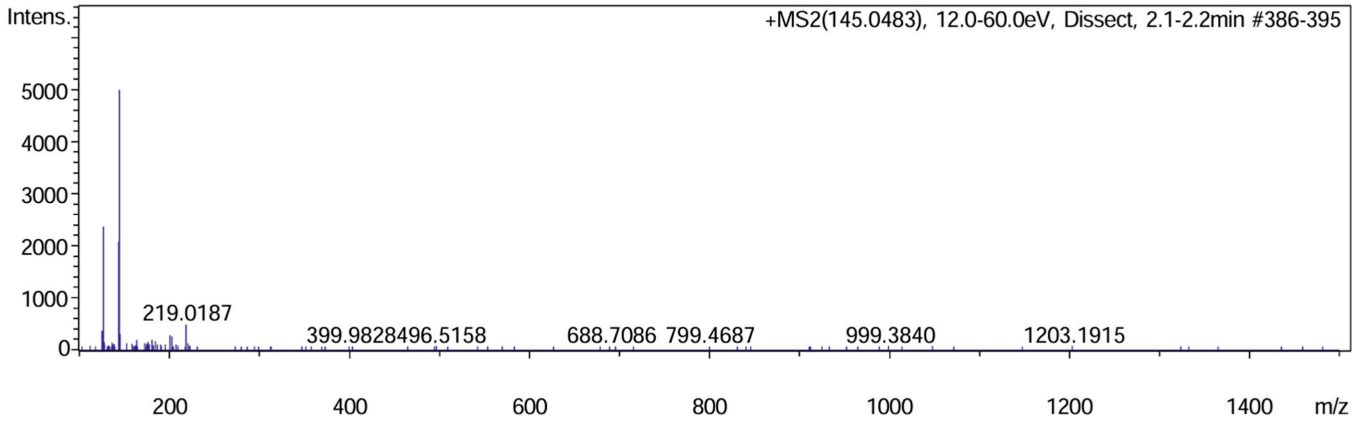


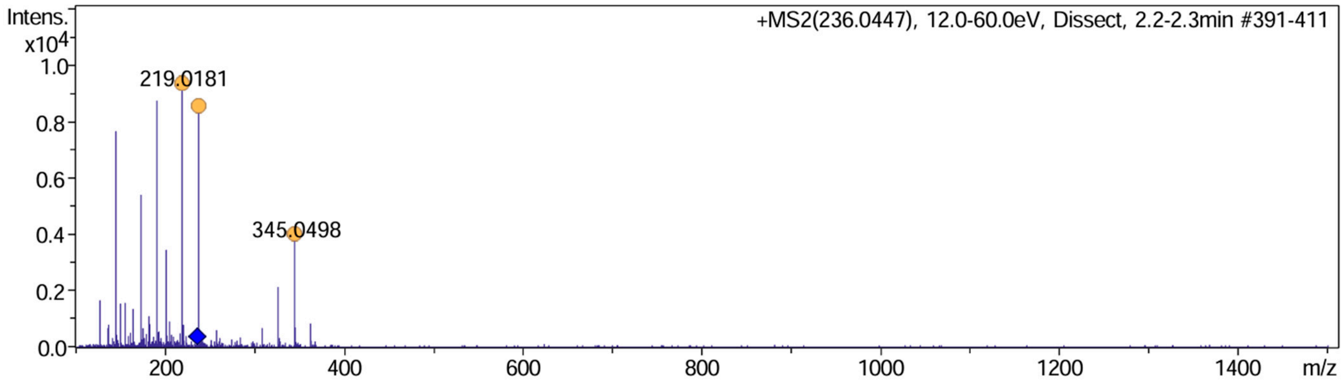


**Cmpd 31, Dissect, 10.6 min**

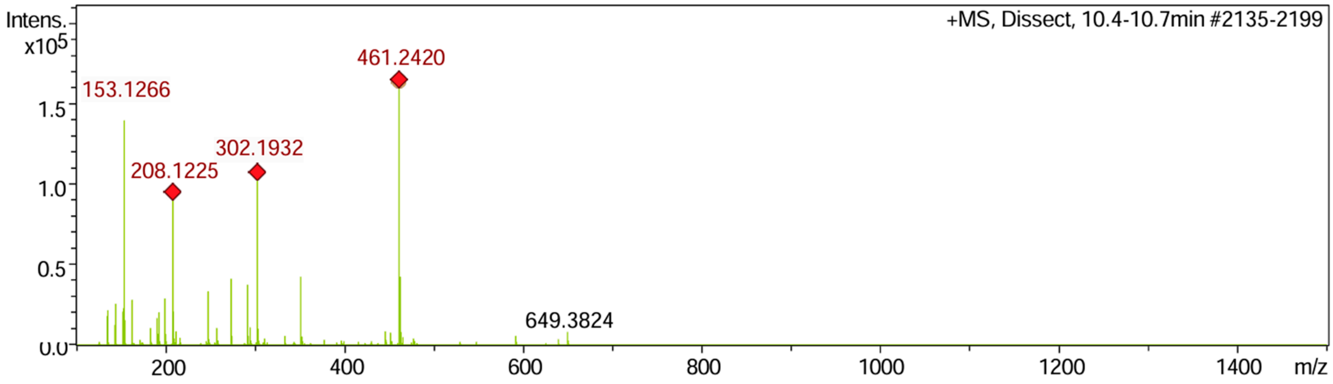

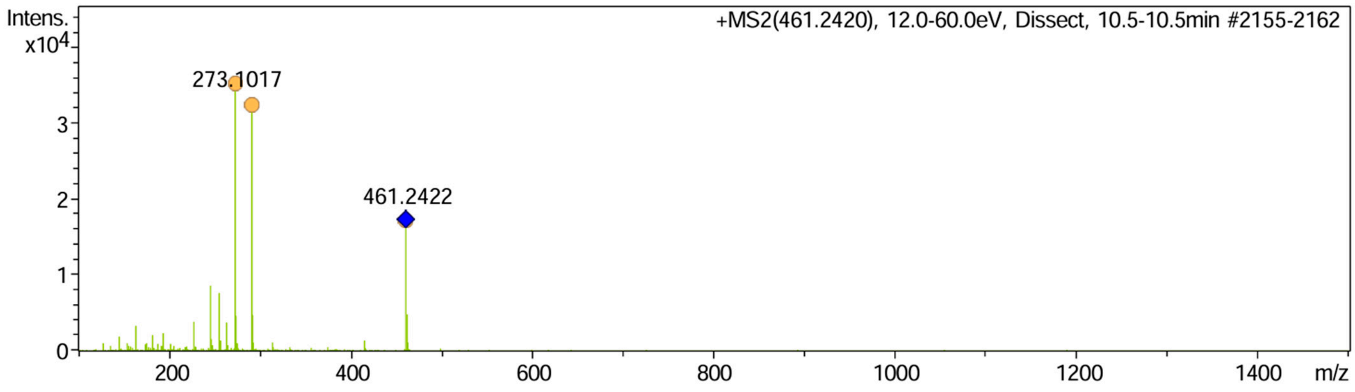

Intens.
x10^4
+MS2(461.2420), 12.0-60.0eV, Dissect, 10.5-10.5min #2155-2162
273.1017
461.2422
m/z


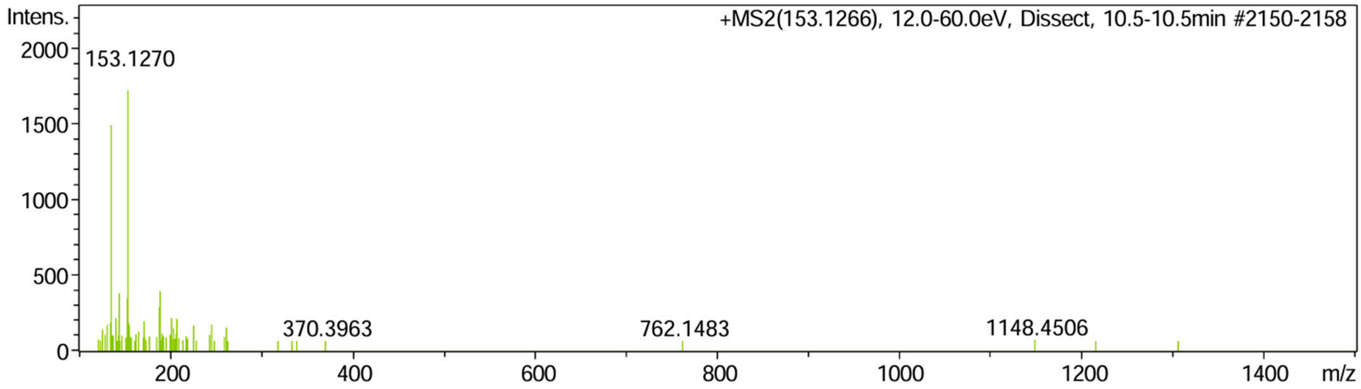

Intens.
+MS2(153.1266), 12.0-60.0eV, Dissect, 10.5-10.5min #2150-2158
153.1270
370.3963
762.1483
1148.4506
m/z


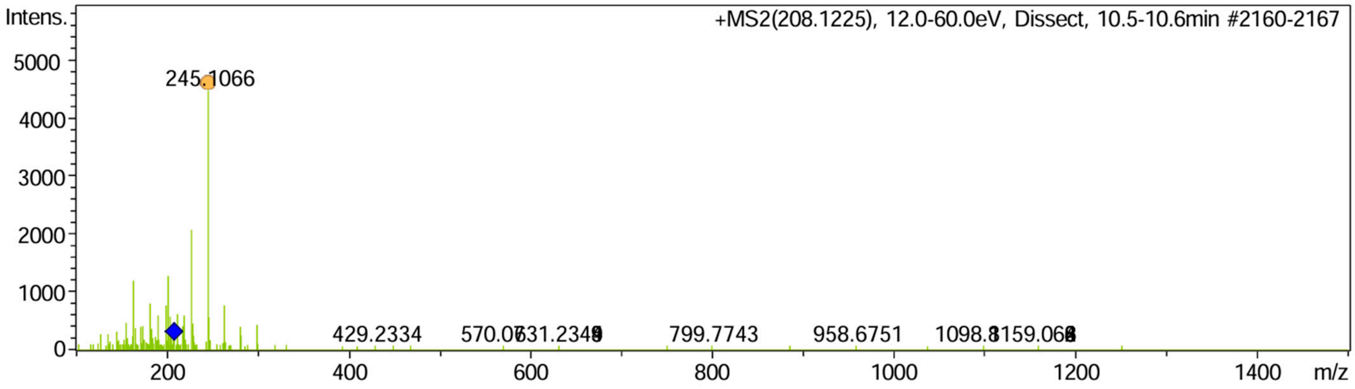

Intens.
+MS2(208.1225), 12.0-60.0eV, Dissect, 10.5-10.6min #2160-2167
245.1066
429.2334
799.7743
958.6751
m/z


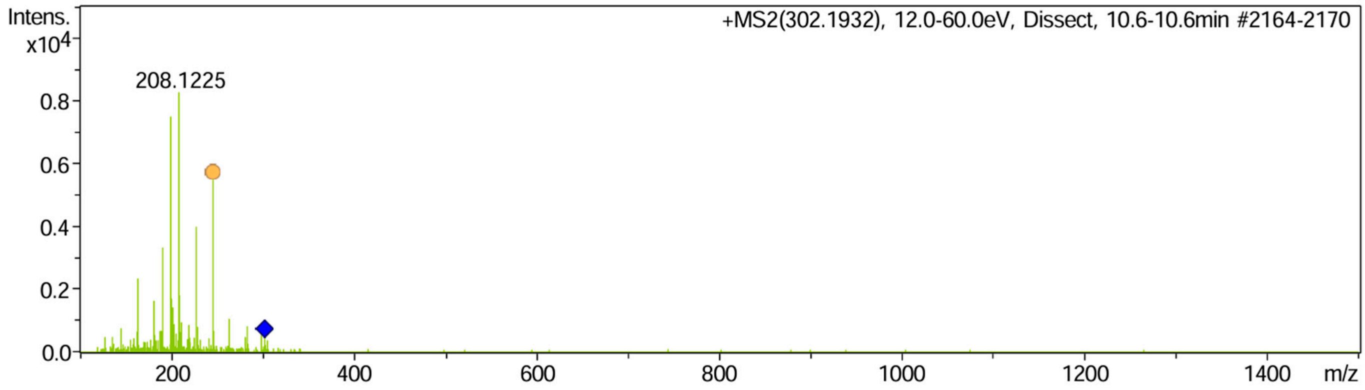

Intens.
x10^4
+MS2(302.1932), 12.0-60.0eV, Dissect, 10.6-10.6min #2164-2170
208.1225
m/z

**Cmpd 34, Dissect, 10.9 min**

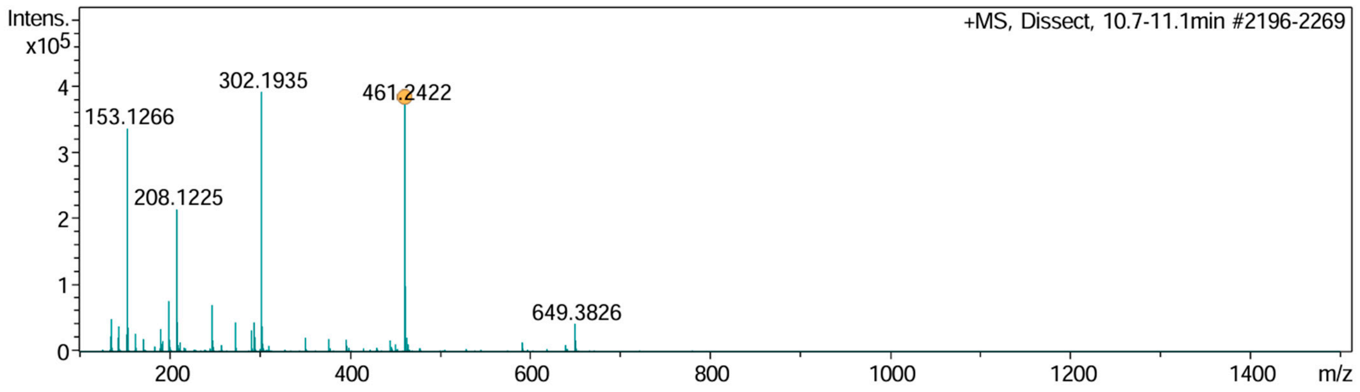


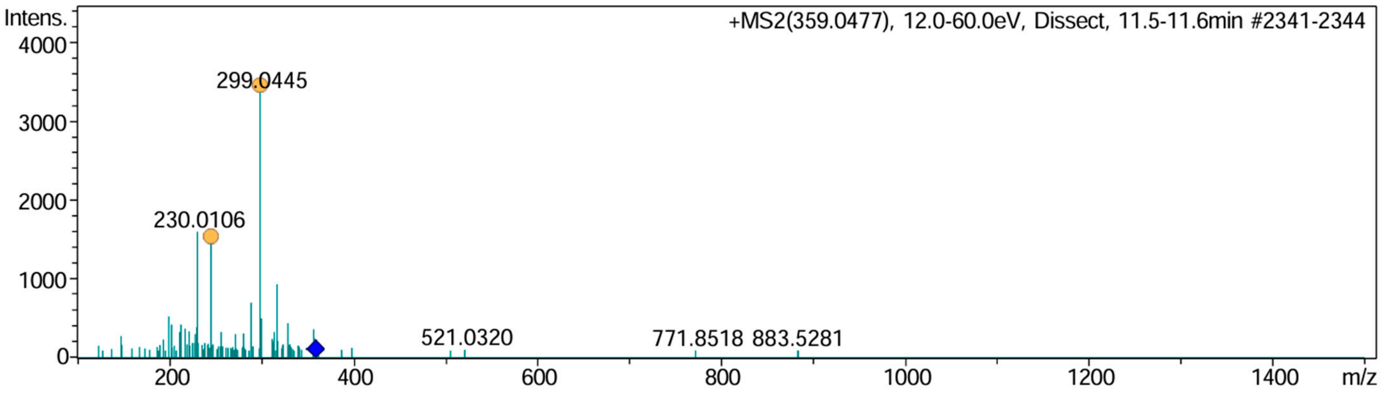


**Cmpd 90, Dissect, 17.4 min**

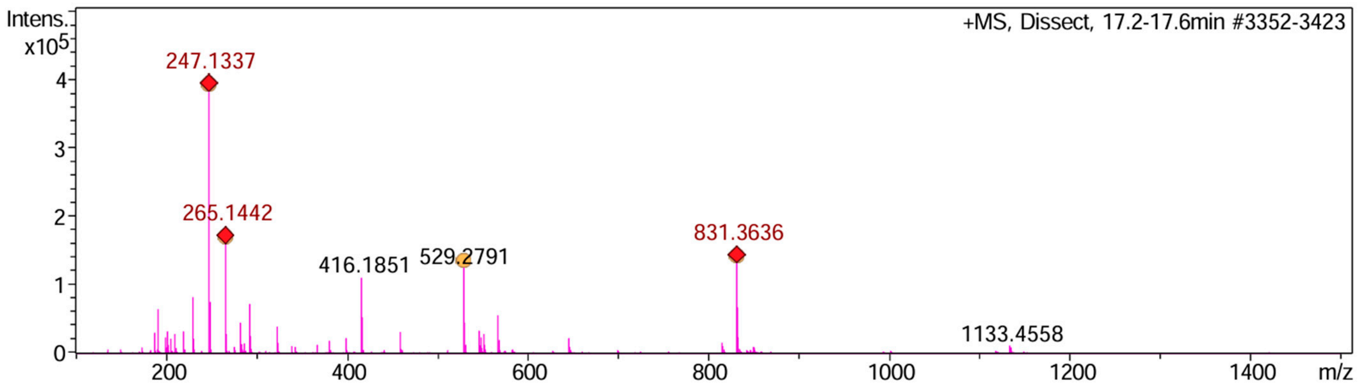

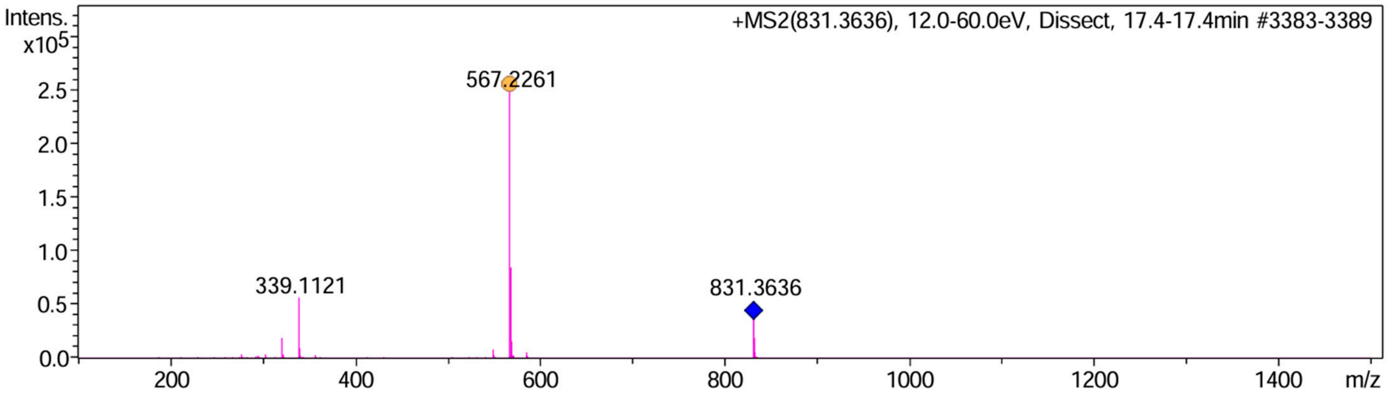


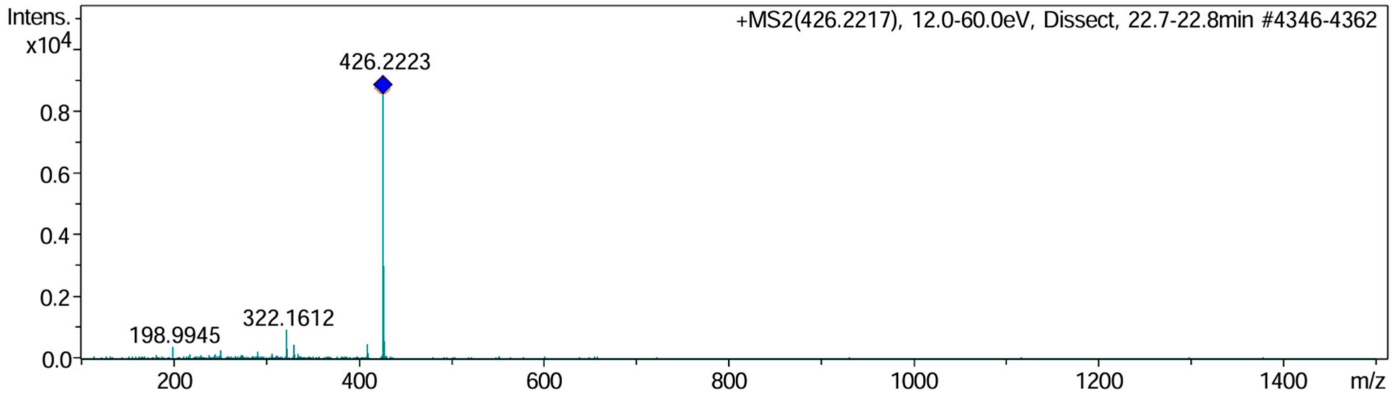


**Cmpd 167, Dissect, 31.5 min**

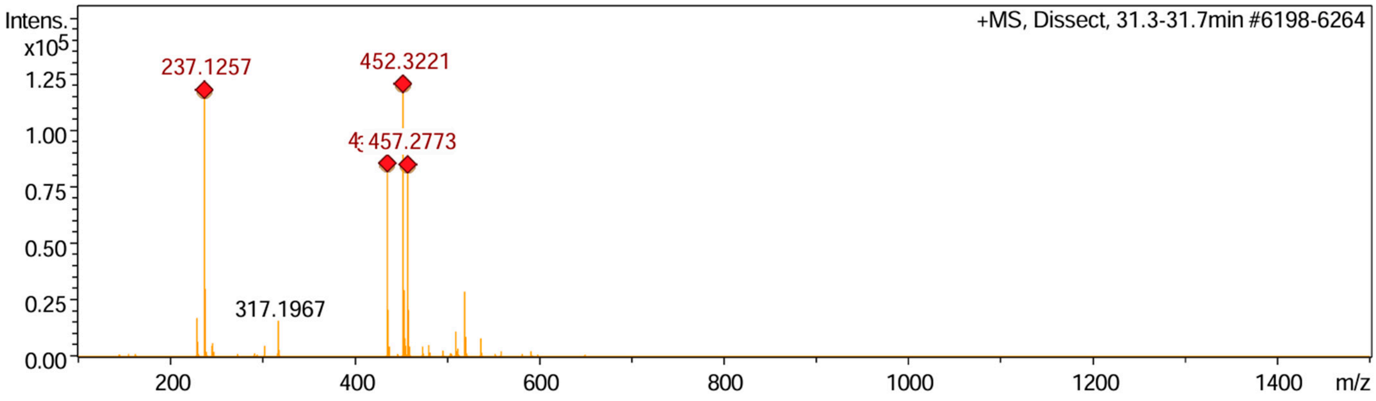


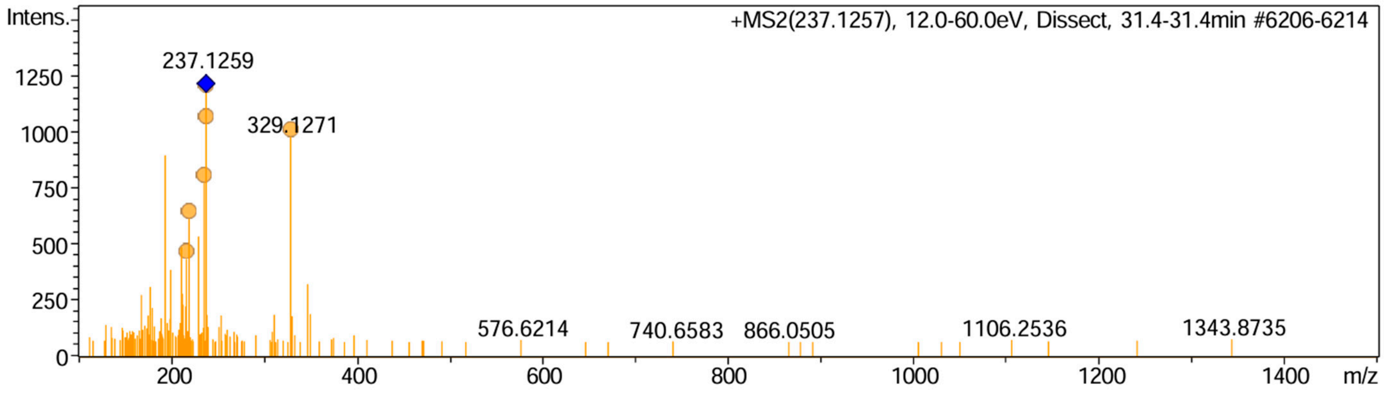

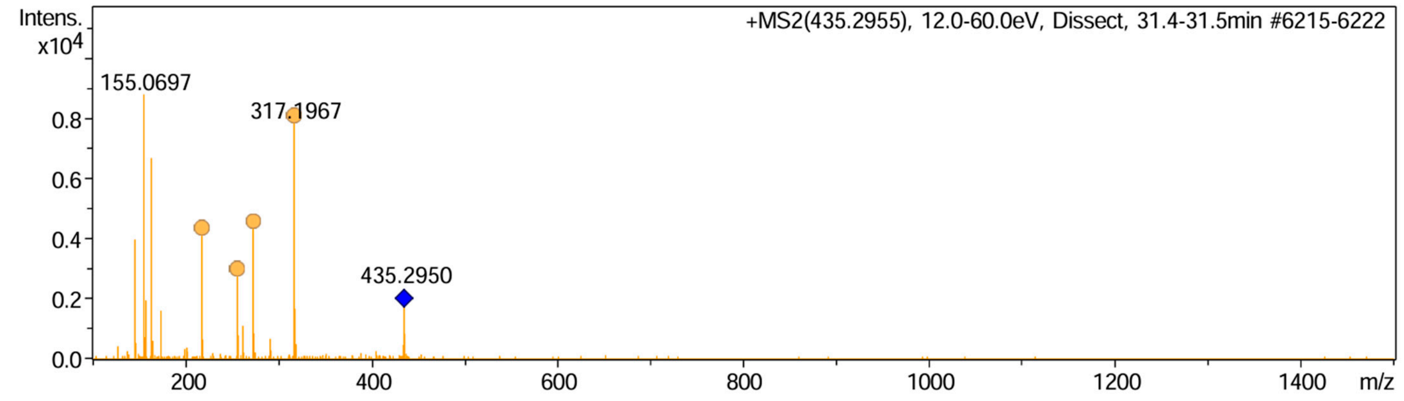
Intens.
x10^4
+MS2(435.2955), 12.0-60.0eV, Dissect, 31.4-31.5min #6215-6222
155.0697
317.1967
435.2950
m/z

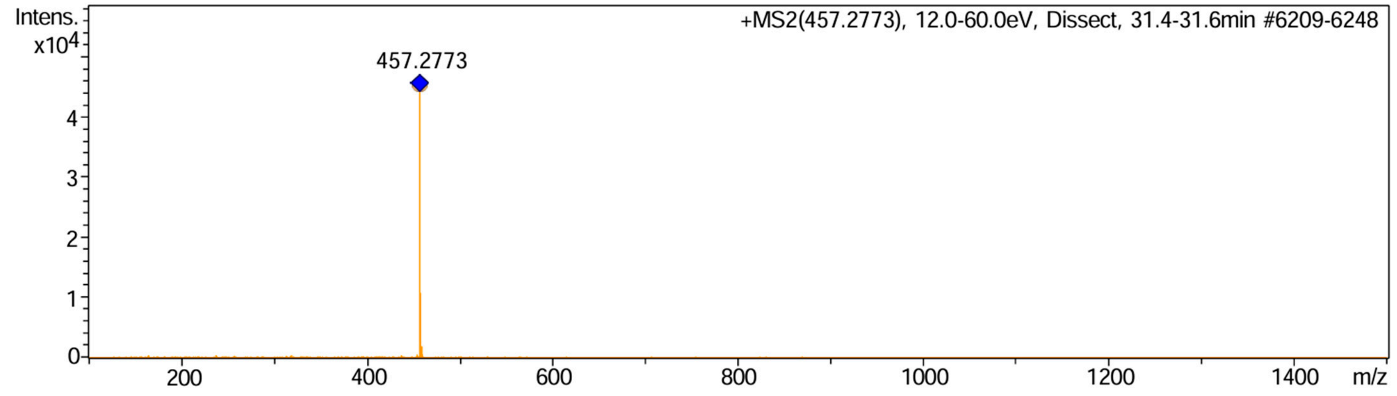
Intens.
x10^4
+MS2(457.2773), 12.0-60.0eV, Dissect, 31.4-31.6min #6209-6248
457.2773
m/z

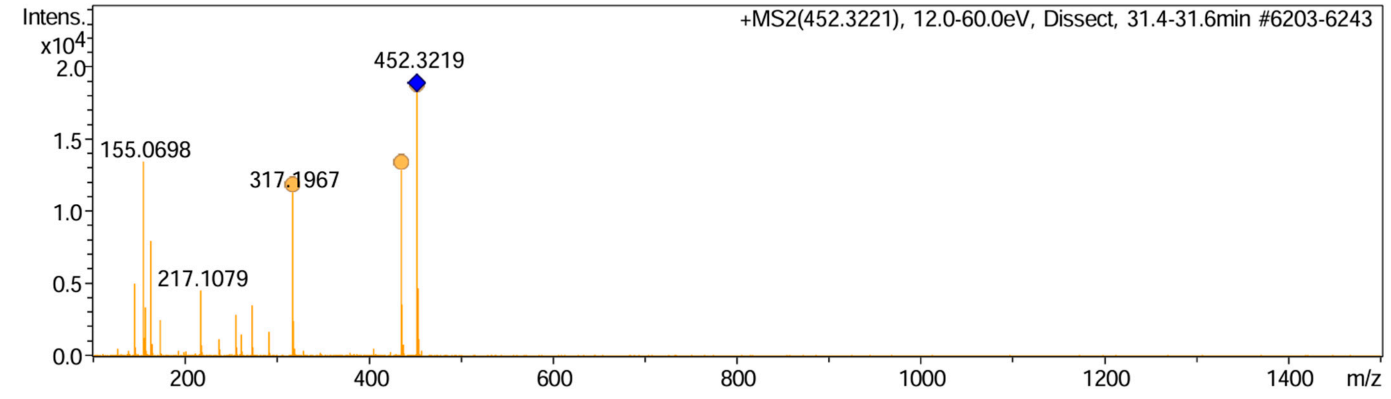
Intens.
x10^4
+MS2(452.3221), 12.0-60.0eV, Dissect, 31.4-31.6min #6203-6243
452.3219
155.0698
317.1967
217.1079
m/z

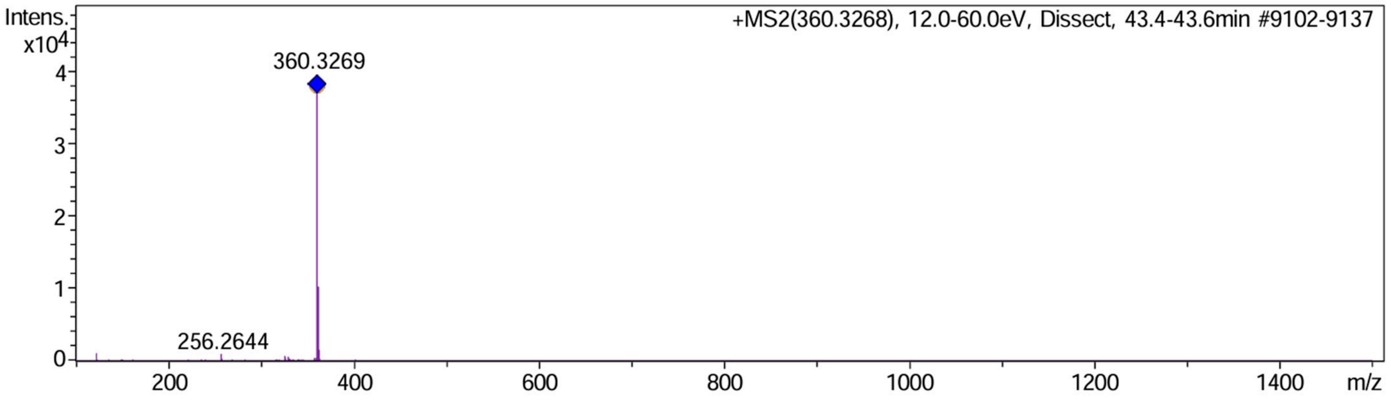
Intens.
x10^4
+MS2(360.3268), 12.0-60.0eV, Dissect, 43.4-43.6min #9102-9137
360.3269
256.2644
m/z

**Cmpd 188, Dissect, 43.9 min**

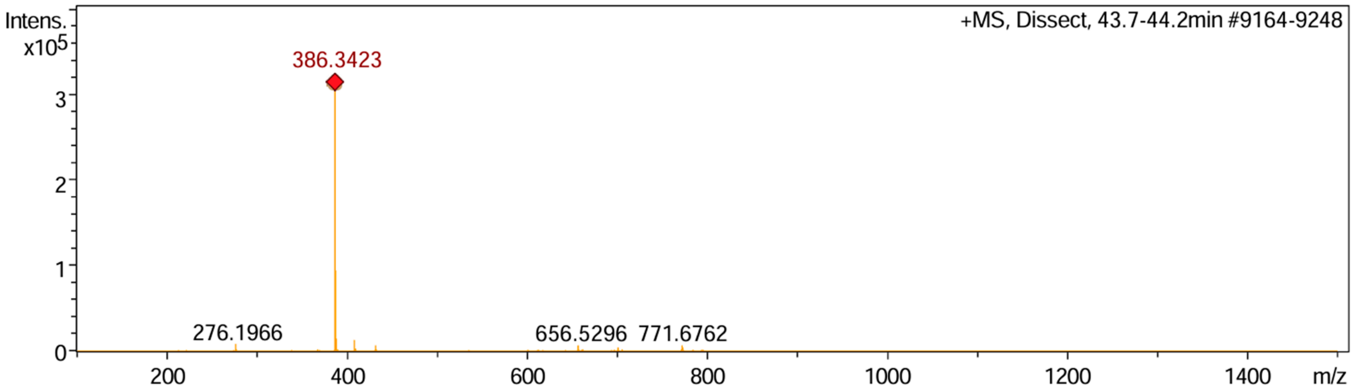


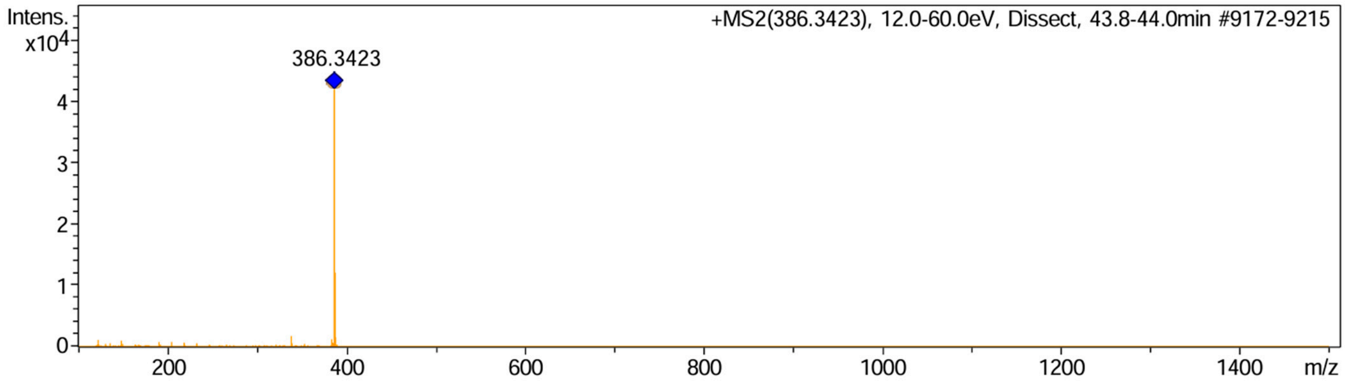

Supplement: Supplementary file 1 [file plants-14-03340-s001.zip › plants-3944175-supplementary.pdf]
